# Plasma-Catalysis of Nonoxidative Methane Coupling: A Dynamic Investigation of Plasma and Surface Microkinetics over Ni(111)

**DOI:** 10.1021/acs.jpcc.2c03503

**Published:** 2022-11-17

**Authors:** Pierre-André Maitre, Matthew S. Bieniek, Panagiotis N. Kechagiopoulos

**Affiliations:** Chemical Processes & Materials Group, School of Engineering, University of Aberdeen, AberdeenAB24 3UE, U.K.

## Abstract

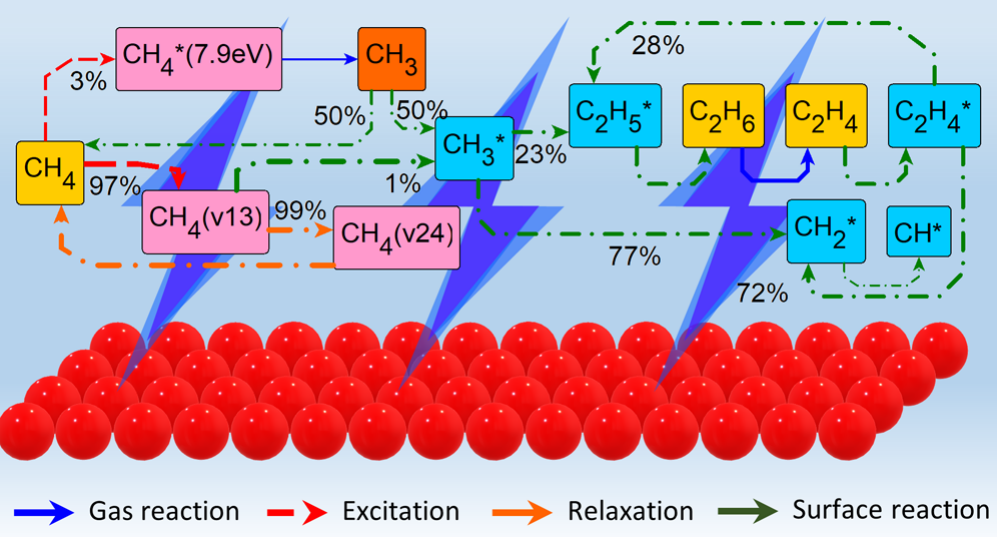

A heterogeneous catalytic microkinetic model is developed and implemented in a zero-dimensional (0D) plasma model for the dynamic study of methane nonoxidative coupling over Ni(111) at residence times and power densities consistent with experimental reactors. The microkinetic model is thermodynamically consistent and is parameterized based on the heats of chemisorption of surface species on Ni(111). The surface network explicitly accounts for the interactions of plasma species, namely, molecules, radicals, and vibrationally excited states, with the catalyst active sites via adsorption and Eley–Rideal reactions. The Fridman–Macheret model is used to describe the enhancement of the rate of the dissociative adsorption of vibrationally excited CH_4_, H_2_, and C_2_H_6_. In combination with a previously developed detailed kinetic scheme for nonthermal methane plasma, 0D simulation results bring insights into the complex dynamic interactions between the plasma phase and the catalyst during methane nonoxidative coupling. Differential turnover frequencies achieved by plasma-catalysis are higher than those of equivalent plasma-only and catalysis-only simulations combined; however, this performance can only be sustained momentarily. Hydrogen produced from dehydrogenation of ethane via electron collisions within the plasma is found to quickly saturate the surface and even promote the conversion of surface CH_3_* back to methane.

## 1 Introduction

Plasma-catalysis is a promising alternative for the direct upgrading of methane into higher-value products as it allows the conversion of methane at much lower temperatures than traditional thermal-catalytic routes.^1,2^ The plasma-activated upgrading of methane in the presence or absence of oxidants has been the subject of numerous experimental investigations in the last two decades. A variety of catalysts and plasma discharges have been studied,^3−20^ confirming the feasibility of the process. Nonetheless, a commonly reported issue is the lack of understanding of mechanistic details leading to observed performances. Plasma-catalysis systems are highly complex, with the plasma affecting the catalyst and vice versa;^21−24^ hence, modeling has been attracting increasing interest to describe some of the complex interactions.

Recent modeling efforts have focused on the impact of vibrationally excited species to explain the experimentally observed, synergistic nature of the plasma and catalyst phases. Engelmann et al.^25^ studied the nonoxidative coupling of methane on various transition metals using a surface microkinetic model. The work predicted a significant increase in the turnover frequency of vibrationally excited methane for the case of low-activity, weakly binding, metals. Loenders et al.^26^ used a similar approach to study the partial oxidation of methane on Pt(111) and found that, even though vibrational excitation of methane and oxygen has a positive effect, it is the plasma-generated radicals that govern the surface chemistry. These studies highlighted the need for kinetic models that encompass the range of species that originate from the plasma phase to describe their impact on and interactions with the catalyst. Nonetheless, most works to date have investigated only initial rates considering a static gas phase, in which populations of vibrationally excited states were dictated by an a priori-decided distribution at a preset vibrational temperature. Dynamic studies of plasma-catalysis, where a plasma-kinetic model is solved concurrently with a catalytic surface one, have only been reported for the production of ammonia.^27−29^ Given that typical discharges used in plasma-catalysis (AC-driven or pulsed) are inherently transient in nature, the dynamic evolution of surface intermediates and gas phase species profiles is of particular interest. Moreover, as recently demonstrated in thermal catalysis by the Dauenhauer group,^30,31^ dynamical effects can potentially be exploited to achieve a higher than the steady-state performance.

In this respect, the objective of this work is to develop a zero-dimensional (0D) dynamic model for the nonoxidative coupling of methane that accounts for both plasma and surface kinetics in detail. Temperature is a determining parameter in thermal catalysis, dictating the activity of the catalyst by influencing its turnover frequency,^32^ while within nonthermal plasmas, it influences gas phase radical reactions and relaxation processes of excited species. As the latter lead to an increase of the gas bulk temperature,^2^ recent plasma-catalysis modeling studies have considered temperatures higher than ambient.^25,26^ To account for this, the impact of temperature on gas and surface processes is investigated in this work in the range from 300 to 600 K at time scales on the order of experimental reactors. Nickel is chosen as a catalyst due to its known capacity to activate C–H bonds and wide application in methane-upgrading processes.^5,10,33−37^ The Ni(111) facet is considered as it is the most common plane in real polycrystalline supported metal catalysts. The performance of the plasma-catalytic reactor at different temperatures is further compared with the equivalent plasma-only and catalysis-only reactors to elucidate the interactions of the two phases. For all cases, a reaction pathway analysis is carried out to elaborate on the profiles of gas and surface species obtained and the turnover frequency (TOF) values achieved. The energy efficiency of all processes is finally analyzed and compared against thermodynamics.

## 2 Methods

### 2.1 Reactor Design and Catalyst Characteristics

**Figure 1:**
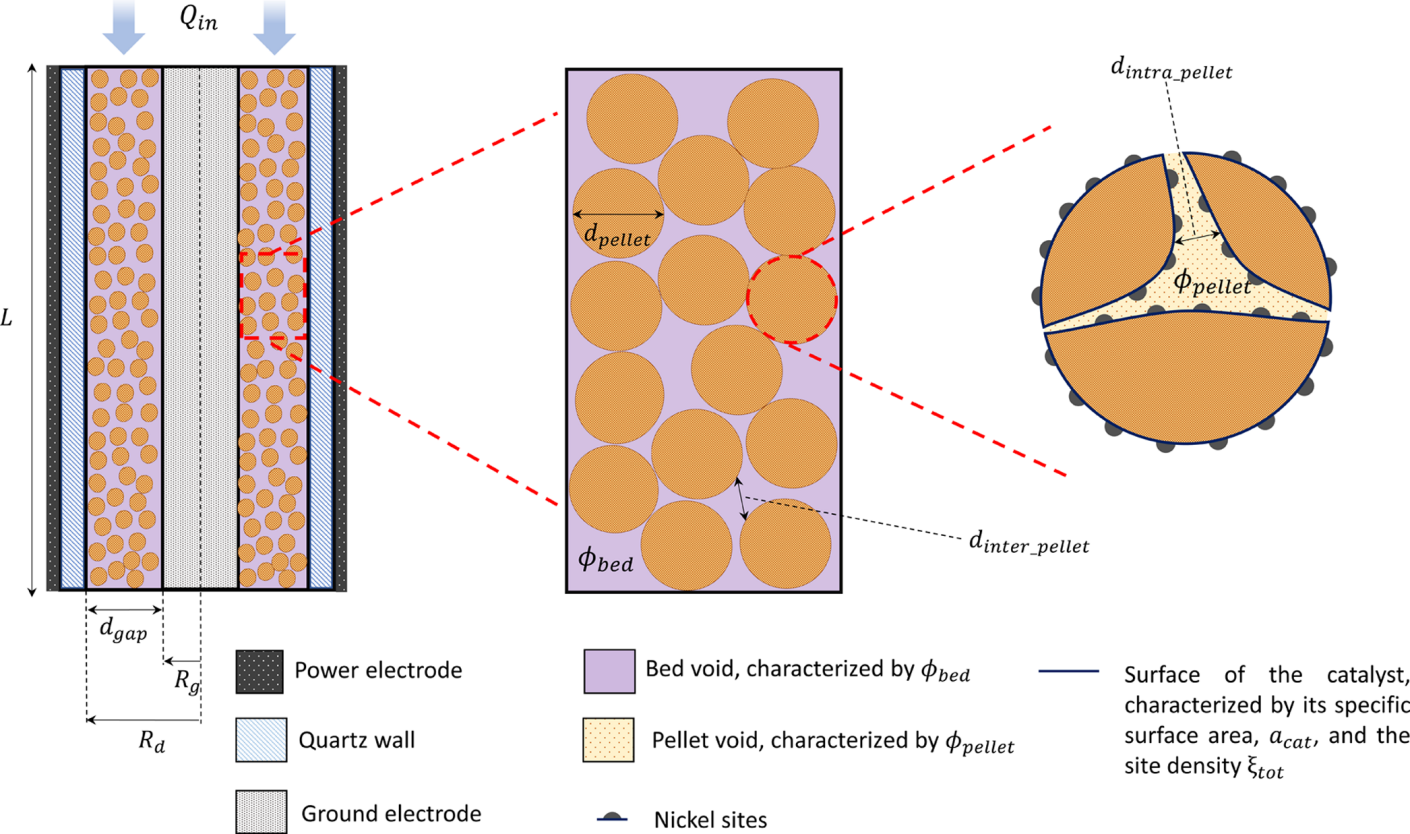
Scheme of the modeled plasma-catalytic reactor, identifying its characteristics across different scales.

Simulations are carried out based on the characteristics of a single-sided dielectric barrier discharge (DBD) reactor (Figure 1). In this design, an internal rod of radius *R*_g_ = 11 mm acts as the ground electrode and is placed concentrically within a quartz tube with internal radius *R*_d_ = 13 mm that acts as the reactor’s external wall and the dielectric of the DBD. The power electrode is positioned externally to the quartz tube. The plasma is formed within the region between the ground electrode and the dielectric of length *L* = 5 mm and the annular gap *d*_gap_ = 2 mm, where the catalyst pellets are also packed. Pure methane is fed at constant flow (see Section 2.2 for the operating conditions considered).

The catalyst characteristics are summarized in Table 1. A site density, ξ_tot_, of 1.8586 × 10^19^ sites·m^–2^ referring to a face-centered cubic (fcc) Ni(111) lattice plane^38^ is used. The specific surface area is of attention, as the value used is 1–2 orders of magnitude lower than typical values of supported catalysts, e.g., on silica and titania^33,39^ or alumina.^10,20,39^ During preliminary simulations, it was seen that the order of magnitude of *a*_cat_ significantly impacts the computational cost of the model, relating to the catalyst’s surface-to-volume ratio. It was hence decided to use a lower specific surface area value to model efficiently long time scales (see Sections 3.1–3.4) and study the impact of higher, more realistic, *a*_cat_ values on energy efficiency in Section 3.5.

**Table 1:** Catalyst Pellet and Bed Parameters Considered in the Simulations

parameter	value
pellet diameter, *d*_pellet_ (mm)	0.4
bed porosity, ϕ_bed_ (−)	0.45
pellet porosity, ϕ_pellet_ (−)	0.5
pellet density, ρ_supp_ (kg·m^–3^)	2300
surface area, *a*_cat_ (m^2^·kg^–1^)	1050
site density, ξ_tot_ (sites·m^–2^)	1.8586 × 10^19^

From the geometrical arrangement of the DBD considered and the catalyst parameters presented, catalyst bed characteristics are estimated (see part 1 of the Supporting Information (SI)) that are necessary to obtain scaling parameters required to solve the set of continuity equations of the model (see Section 2.3).

### 2.2 Operating and Initial Conditions

Different operating conditions are considered to study the nature of interactions between plasma and the catalyst and compare performances with equivalent plasma-only and catalysis-only cases. For all cases, the inlet methane density, *n*_β_0__, at different temperatures is the same, resulting in inlet reactor pressures, *P*_0_, that range from 1 bar at 300 K to 2 bar at 600 K (Table 2). A flow, *Q*_in_, of pure methane is considered for all cases, with the reactor residence time, τ, obtained from
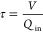
1

**Table 2:** Operating Conditions Considered^a^

case	*T*_0_ (K)	*P*_0_ (bar)	*P*_applied_ (W)	SEI (MJ·m^–3^)	τ (s)	*P*_d_ (W·m^–3^)
plasma-only	300	1.00	50	10	0.151	6.6 × 10^7^
	400	1.33				
	500	1.67				
	600	2.00				
catalysis-only	300	1.00	0	0	0.109	0
	400	1.33				
	500	1.67				
	600	2.00				
plasma-catalysis	300	1.00	50	10	0.109	9.1 × 10^7^
	400	1.33				
	500	1.67				
	600	2.00				

a For all cases, the initial methane density and flow rate are *n*_*β*_0__ = 2.45 × 10^25^ m^–3^ and *Q*_in_ = 5 × 10^–6^ m^3^·s^–1^.

In the above, *V*_reac_ and *V*_void–total_ are used as volumes for plasma-only and (plasma)-catalysis cases, respectively (see SI part 1), resulting in larger residence times for the former (Table 2).

For plasma cases, a power, *P*_applied_, of 50 W, within the range typically applied in DBD reactors,^5,13,40,41^ is used, resulting in a specific energy input, SEI, of 10 MJ·m^–3^. The very fast dynamics of plasma events coupled with surface reaction dynamics lead to a numerically stiff problem that is challenging to solve and computationally expensive. To facilitate the solution of the current model, the power density input, *P*_d_, is assumed to be constant (see the energy continuity equation of the electrons in Section 7) and not a sequence of pulse and afterglow periods commonly considered in 0D plasma-only DBD models.^40,42,43^ However, as elaborated in Maitre et al.^44^ and the literature,^40,42,45^ methane conversion is mainly driven by the amount of energy inputted per unit of plasma volume (the SEI). It is, therefore, considered that the approach followed allows the qualitative investigation of plasma–catalyst interactions. The power density input, *P*_d_, is thus inferred from the SEI and the residence time of the reactor
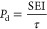
2

Simulations are executed for a time equal to 10 τ, with the gas phase containing initially only pure methane and the catalyst surface considered empty. Catalysis-only cases, in addition to the surface reaction network, consider the complete gas phase reaction network from the plasma model, however, at zero power input. The trajectories and values obtained at the end of simulations of densities and coverages for all catalysis cases are subject to the initial state of the catalyst surface. Considering that an empty surface is assumed to initialize these simulations, results can be considered as describing the startup of the reactor or the evolution of the catalyst surface in a pulse-afterglow operation, where the afterglow period is long enough for the surface to alter to a degree it can be considered equivalent to empty.

### 2.3 Continuity Equations and Scaling Parameters

The continuity equations used to model the various reactor types are developed based on certain considerations elaborated in the following, with the rates of the various processes scaled accordingly. For the selected catalyst characteristics and operating conditions, the Debye length is larger than the internal catalyst pore diameter (see SI part 2). Consequently, the plasma and electron collisions are assumed not to occur within the pores of the catalyst pellet.^22^ Electronically excited states and ions are also assumed not to be present in the catalyst pores due to their very short lifetimes and low densities.^2,21,46^ Radicals and vibrationally excited species have longer lifetimes and higher densities^2,44,47^ and hence are considered to be able to enter the catalyst pores and have access to the total catalyst surface area, *A*_cat_.

For the charged and the neutral species, denoted with subscripts α and β, respectively, the mass balance equations read
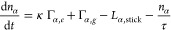
3
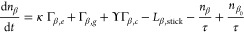
4where Γ*_e_* (m^–3^·s^–1^), Γ_g_ (m^–3^·s^–1^), and Γ_c_ (m^–2^·s^–1^) are the species net formation rates by electron collisions, gas phase reactions (not involving electrons), and catalytic reactions, respectively. κ(−) is a scaling factor (see Table 3) that accounts for the electron processes not taking place inside the pores of the catalyst. *L*_*a*/β,stick_ (m^–3^·s^–1^) are the rates of sticking of ions/neutral species,  (m^–3^·s^–1^) are the reactor outlet flow rates, and  (m^–3^·s^–1^) is the inlet flow rate (relevant only for the feed methane). The catalytic processes are scaled by a factor *Υ* (m^–1^), representing the total surface of the catalyst over the total volume available for gas phase processes (Table 3). This parameter is set to 0 for plasma-only cases.

**Table 3:** List of Scaling Parameters and Respective Values for the Different Cases Simulated

scaling parameter	case	calculation	value
κ (−)	plasma-only		1
	catalysis		0.62
ψ_α_ (m^–1^)	plasma-only		1.00 × 10^3^
	catalysis	;	1.83 × 10^4^
ψ_β_ (m^–1^)	plasma-only		1.00 × 10^3^
	catalysis		1.38 × 10^3^
Υ (m^–1^)	catalysis		9.18 × 10^5^

The sticking rates of ions and neutral species follow the same formalism as in Maitre et al.^44^
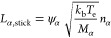
5
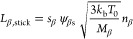
6where *M* (kg) is the mass of the species considered. The sticking probabilities, *s*_β_ (−), considered in Maitre et al.^44^ are also used in this work, while for ions a value of 1 is assumed. The sticking rates are scaled by a factor ψ (m^–1^), which corresponds to the ratio of the surface where sticking can occur over the gas volume available to the species, depending on both the reactor case and the species type (see Table 3). For the catalysis cases, it is assumed that the reactor walls and the external surface of the pellets are accessible to ions (see ψ_α_ in Table 3), while neutral species can only stick on the reactor walls, as catalytic reactions can take place on the catalyst surface (see ψ_β_ in Table 3).

Finally, the electron energy continuity equation reads
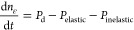
7where *n*_ε_ = *n*_e_ε is the mean electron energy density, *P*_d_ is the power input density discussed previously, *P*_elastic_ is the power density lost by the electrons in elastic collisions in the gas phase, and *P*_inelastic_ is the power density lost in inelastic collisions in the gas phase (namely, in excitation, ionization, and dissociation processes presented in Maitre et al.^44^).

The model is implemented in the Plasimo software,^48^ while the electron energy distribution function (EEDF) is obtained using the BOLSIG+ solver,^49^ via the cross sections used in Maitre et al.^44^ The rate constants of all electron collision processes are derived by integration of the collision cross sections over the EEDF following procedures elaborated in a previous work.^44^

### 2.4 Surface Reaction Network

The microkinetic model describing the surface reaction network is developed under the assumption that only neutral species can interact with the catalyst active sites. As discussed, given their low densities and short lifetimes, charged species are assumed not to be present in the catalyst pores. Moreover, the energies of ions at plasma-catalysis conditions are typically lower than those required to induce interactions with the surface.^50^ Negative surface charging, as revealed in recent density functional theory (DFT) studies related to CO_2_ dissociation, can enhance catalyst activity;^51^ hence, when similar data become available for CH_4_, it would be of value to account for such effects, if present, in global modeling studies. All reactions are considered elementary and reversible, with forward and reverse steps implemented separately. The rate of a given reaction is calculated following the law of mass action. Forward and reverse rate constants are calculated under constraints that ensure thermodynamic consistency (see SI part 3). Adsorption of species can be either molecular (no bond is broken and one site is occupied) or dissociative (two sites required and bond cleavage occurs). Surface reactions follow either the Langmuir–Hinshelwood (two adsorbed species react) or the Eley–Rideal (collision of a gas phase species with a surface one) formalisms. Pre-exponential factors of adsorptions and forward Eley–Rideal reactions are estimated using collision theory. Forward pre-exponential factors of Langmuir–Hinshelwood reactions are obtained using transition state theory estimates. Reverse pre-exponential factors for all reactions are obtained from entropic consistency. Molecular (nondissociative) adsorptions are considered nonactivated. Forward activation barriers for dissociative adsorptions and Langmuir–Hinshelwood reactions are obtained using the unity bond index–quadratic exponential potential (UBI-QEP) method. The method is selected, instead of using directly kinetic data on Ni(111), as it provides a straightforward manner to evaluate the catalytic activity of a range of metals based on a set of well-chosen catalyst descriptors (similar to the use of scaling relationships and/or Brønsted–Evans–Polanyi relationships, whose availability, though, for all reactions of interest is not always guaranteed). Forward activation barriers for Eley–Rideal reactions are obtained using the Polanyi–Semenov correlation proposed by Krylov.^52^ Reverse activation barriers for all reactions are obtained from enthalpic consistency (see SI part 4).

Based on the low gas phase densities obtained in our previous work,^44^ the adsorption of C_3_ species is omitted. Methane, ethane, and hydrogen are known to adsorb dissociatively,^38^ resulting in a total of 10 surface species (CH_3_*, CH_2_*, CH*, C*, C_2_H_5_*, C_2_H_4_*, C_2_H_3_*, C_2_H_2_*, C_2_H*, H*) being included in the surface model. The microkinetic model is parameterized based on the heats of chemisorption of these species, χ_*i*_, which act as catalyst descriptors and are taken from the DFT study of Han et al.^38^ (see Table 4). For all heat of chemisorption values, the Ni(111) lattice and the most stable position with the lowest binding energy are used. Coverage-dependent energetics due to lateral interactions of adsorbates are not considered given the overall low coverages obtained (see Section 3).

**Table 4:** Heat of Chemisorption Values (eV) of the Considered Surface Species on Ni(111)^38^

species	CH_3_*	CH_2_*	CH*	C*	C_2_H_5_*	C_2_H_4_*	C_2_H_3_*	C_2_H_2_*	C_2_H*	H*
χ_*i*_ (eV)	2.02	4.44	6.66	7.03	1.60	0.86	3.07	2.62	5.34	2.68

A total of 58 reversible processes are included in the model, which are presented in detail in the SI (parts 5–7). In brief, ten molecular adsorptions and three dissociative adsorptions, along with their reverse processes, are considered. Once adsorbed, the hydrocarbon species can dehydrogenate via seven processes as follows
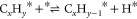
or undergo hydrogen transfer via nine processes according to
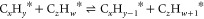
Carbon coupling of species at the surface of the catalyst is possible via two routes
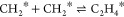

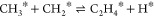
Finally, 27 Eley–Rideal processes leading to hydrogen abstraction/addition between surface species and gas phase radicals are included
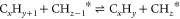
Only adsorbed species containing one carbon atom are considered for Eley–Rideal processes (*z* = 1, 2, 3), whereas the colliding radicals can contain up to three carbon atoms (*x* = 1, 2, 3).

For all vibrationally excited states, equivalent surface reactions to those of the respective ground states are considered. Indeed, various studies have demonstrated the strong mode specificity of CH_4_ dissociative adsorption on metal surfaces, with vibrational excitation enhancing considerably the adsorption rate in comparison to that of the ground state.^53−55^ The contribution of vibrational excitation energy in overcoming the activation barrier of the dissociative adsorption of CH_4_, C_2_H_6_, and H_2_ is considered according to the Fridman–Macheret model (see SI part 4). Molecular adsorptions and Eley–Rideal reactions of vibrationally excited states are assumed to proceed at the same rate as that of the ground state. As nondissociative adsorptions proceed with no barrier, the vibrational energy of excited states is assumed to be dissipated upon adsorption.^50^ For Eley–Rideal reactions, preliminary simulations revealed the dominant path to be also barrierless, so for simplicity, a similar assumption was made. Electronically excited states and ions, due to their very low densities and extremely short lifetimes,^44^ are considered not to interact with the catalyst surface.

Finally, the total balance of active sites is considered
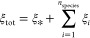
8where ξ_*_ is the vacant site surface density and ξ_*i*_ is the site surface density that is occupied by surface species *i*.

## 3 Results and Discussion

### 3.1 Methane and Final Product Densities

**Figure 2:**
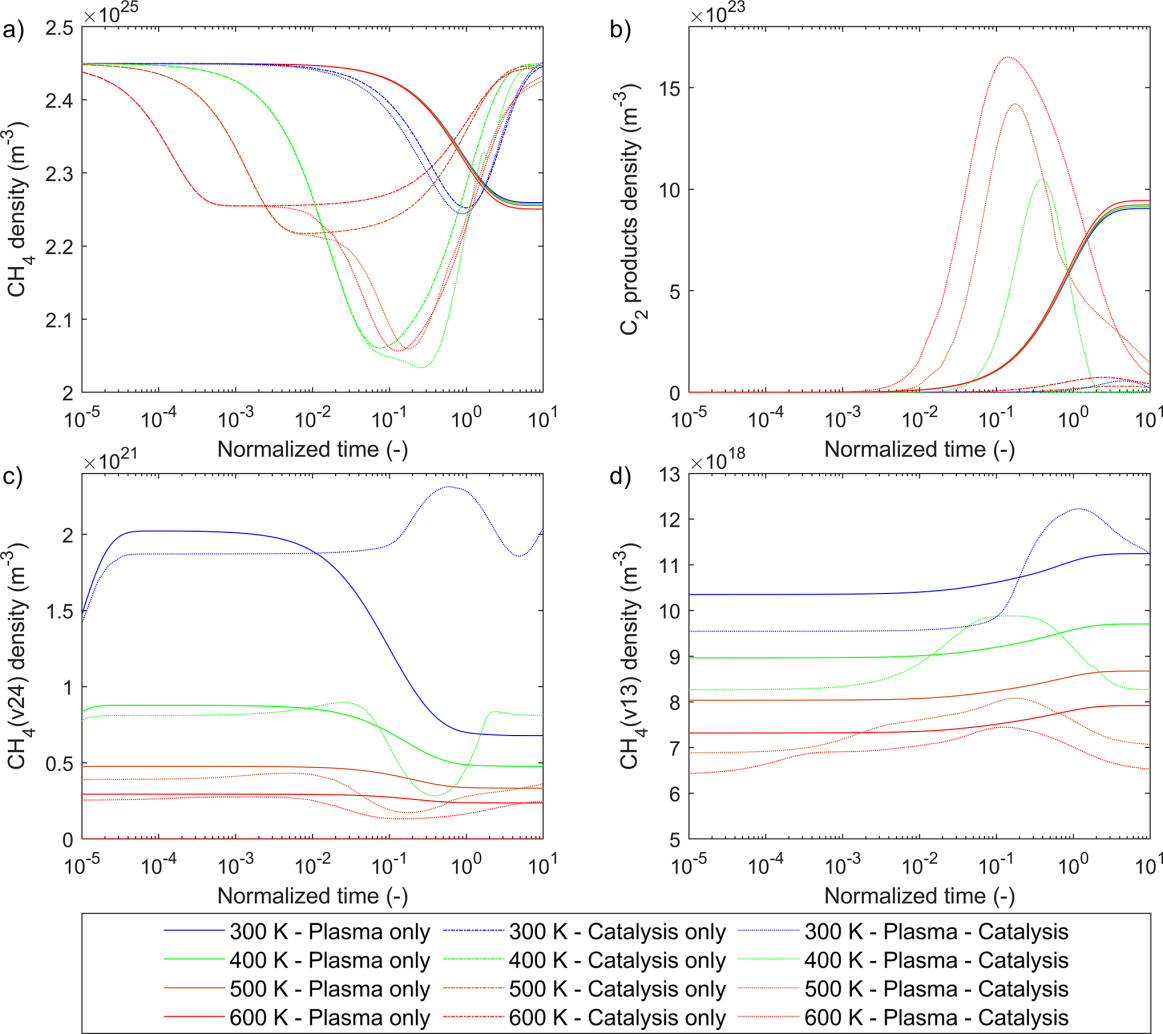
Gas species number densities (m^–3^) against normalized time in the logarithmic scale for the different cases simulated. (a) CH_4_, (b) C_2_ species, (c) CH_4_(ν2, 4), and (d) CH_4_(ν1, 3). Figure S1 in the SI (part 8) reproduces the same data in the linear scale to demonstrate the approach to steady state.

The densities of CH_4_ and its excited states, and of C_2_ products, for all temperatures and reactor cases studied are presented in Figure 2. The results are plotted against normalized time (*t*/τ), as the residence time differs between plasma-only and (plasma-) catalysis cases. Figure 2 presents results in the logarithmic scale to put focus on the initial highly transient response of the variables, while Figure S1 in the SI (part 8) presents the same results in a linear scale to demonstrate the approach to steady state. Density profiles for the main radicals are also provided in the SI (part 9).

Methane and C_2_ species densities exhibit similar trends in plasma-only simulations (solid lines in Figure 2), reaching a steady state within a simulation time of approximately 5 τ. As the density of CH_4_ decreases, that of C_2_ species increases, in line with the results presented previously.^44^ Temperature variation within the range studied has overall a negligible impact, in agreement with methane thermal decomposition occurring over 800 K.^56^ The small increase in the conversion of methane observed with rising temperature is attributed to the acceleration of the step H + CH_4_ → CH_3_ + H_2_, whereas plasma processes remain largely unaffected.

Catalysis-only cases (dashed lines in Figure 2) exhibit a much more transient response, characterized for all temperatures by the density of methane initially decreasing and subsequently increasing. The drop in CH_4_ density is due to its adsorption on the surface of the catalyst, which results in a gradual saturation of the catalyst active sites (see Section 3.2). As the surface coverage of CH_x_* fragments rises, desorption reactions are promoted, resulting in a decrease of the net rate of methane adsorption. Adsorption and desorption reactions of methane eventually equilibrate at approximately 10 τ, with methane conversion achieved being very low from that time onward (≤1%). At 300 K, the adsorption rate of methane is slow, as indicated by the less pronounced decrease in CH_4_ density in comparison to the other catalysis-only cases (Figure 2-a). At 400 K, methane adsorption accelerates, while its desorption is slower than at 500 and 600 K, resulting in the lowest density of methane achieved. C_2_ species densities are at all times very low, given the low conversion of methane. Qualitatively, these results agree with the work from Okolie et al.,^57^ who studied nonoxidative coupling of methane over nickel for temperatures lower than 773 K and found methane conversions below 1.2%.

The plasma-catalysis responses (dashed-dotted lines in Figure 2) initially follow similar trends to those of the catalysis-only cases (Figure 2a). However, at 400 K, and especially at 500 and 600 K, the lower methane densities achieved evidence a clear effect of plasma interaction with the catalyst. Unlike the equivalent catalysis-only cases where adsorption and desorption of methane balanced over time, during plasma-catalysis simulations, the excited states of methane and the radicals originating from the gas phase enhance the conversion of methane (discussed in further detail in Section 3.4). This is confirmed by the densities of C_2_ molecules (Figure 2b), which reach peak values two times higher than the sum of plasma-only and catalysis-only cases. As with the density of methane, a higher production of C_2_ is achieved at 500 and 600 K in comparison to that at 400 K. This confirms the ability of plasma to increase the differential turnover frequency of a catalyst (see Section 3.3) and lead to synergistic effects at temperatures too low for thermal catalysis activity (but above 400 K in our simulations). Nonetheless, as observed also for the catalysis-only cases, the methane density increases again in the later stages of the simulations due to the increased desorption of CH_4_. These trends are a consequence of the high coverage of H* surface species originating from H_2_ and H in the plasma phase and are discussed in detail in Section 3.2, elaborating on surface densities and rates.

The populations of CH_4_(ν2, 4) and CH_4_(ν1, 3) obtained with plasma-only and plasma-catalysis scenarios are presented in Figure 2c,d, respectively. Given the absence of plasma, there is no production of excited states in catalysis-only cases. For both plasma cases, the densities of CH_4_(ν2, 4) and CH_4_(ν1, 3) are on average 4 and 5 orders of magnitude lower than those of ground-state CH_4_ and well below the peak values reported in Maitre et al.^44^ and in other studies involving DBDs.^40^ These results are due to the discharge being simulated as a homogeneous plasma, with 2–3 orders of magnitude lower power density inputted than generally applicable in pulse and afterglow models.^40,43,45^ Nonetheless, qualitative differences can still be discerned based on reactor case and temperature. Specifically, lower average densities of excited states are predicted with higher temperatures and for plasma-catalysis cases on account of the higher rates of vibrational–translational (VT) and vibrational–vibrational (VV) processes and faster adsorption rates on the catalyst (these effects being more pronounced for CH_4_(ν1, 3)). Interestingly, the density of CH_4_(ν2, 4) during plasma-catalysis and above 300 K follows a similar trend to that of ground-state CH_4_, passing through a minimum, while the opposite profile is observed for CH_4_(ν1, 3). These trends can be explained by a reduction of the rate of the VV process CH_4_(ν1, 3) + CH_4_ → CH_4_(ν2, 4) + CH_4_(ν2, 4) due to the consumption of CH_4_ on the catalyst, highlighting an effect the catalyst has on plasma species.

Finally, the selectivities of products are not significantly influenced by the temperature and are therefore presented in the SI (part 10). For plasma-only cases, selectivities of 26% for ethane, 50% ethylene, and 18% acetylene are obtained for all temperatures (in line with the results of Maitre et al.^44^ for a 10 MJ·m^–3^ SEI). For catalytic cases (with or without plasma), the product formation is dominated by surface chemistry. Ethane accounts for nearly 100% of the products, with only traces of ethylene (≈0.0005%) and C_3_ species (≈0.0008% propane and ≈0.001% propylene), results that compare well with the experimental literature of nonoxidative methane coupling over nickel.^36,57^

### 3.2 Surface Species and Rates

**Figure 3:**
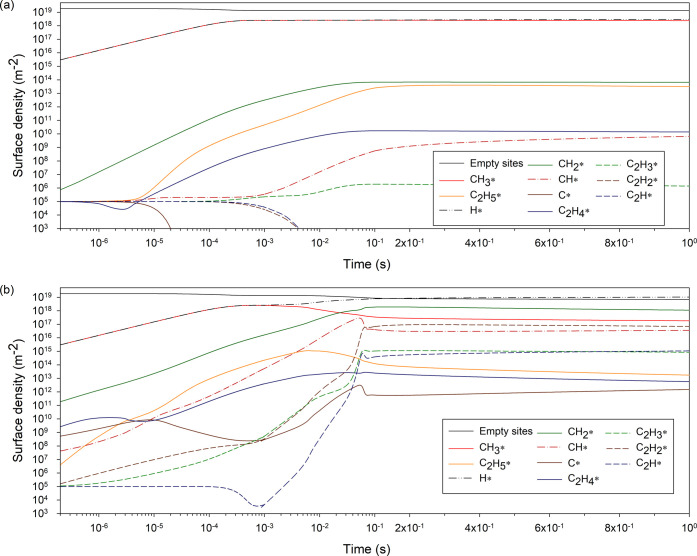
Surface density of adsorbed species (m^–2^) over time (s) for (a) catalysis-only and (b) plasma-catalysis cases, at 500 K. For times below 0.1 s, the *x*-axis is logarithmic, and linear afterward.

**Figure 4:**
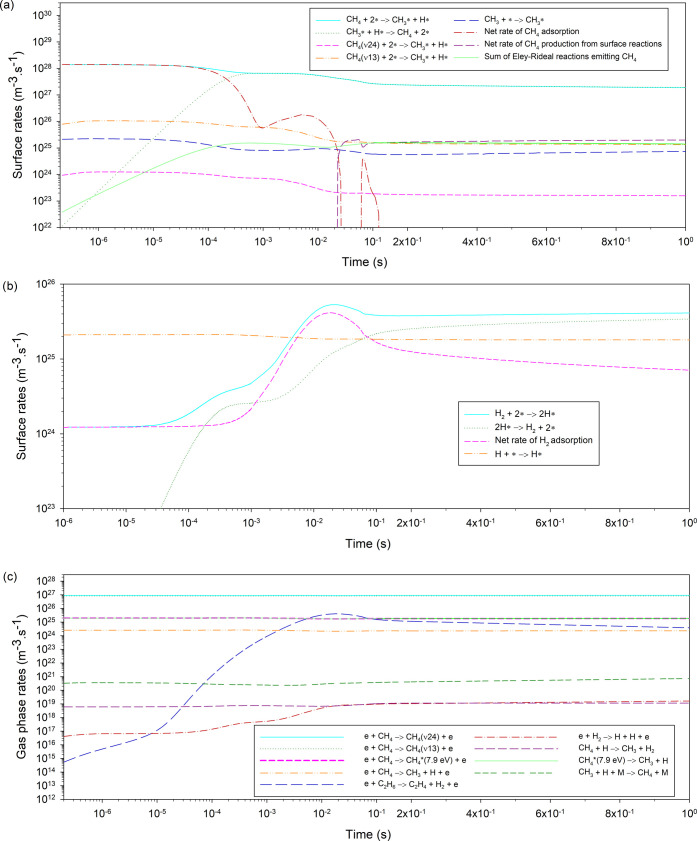
Most significant rates (m^–3^·s^–1^) over time (s) for the plasma-catalysis case at 500 K, (a) involving methane at the surface, (b) involving hydrogen at the surface, and (c) in the gas phase. For times below 0.1 s, the *x*-axis is logarithmic, and linear afterward.

Figure 3 presents the evolution of surface densities of species during catalysis-only (panel a) and plasma-catalysis (panel b) cases at 500 K. This temperature is selected to elaborate on plasma–catalyst interactions as it is the lowest where significant synergistic effects were observed (Section 3.1). Other temperatures exhibit qualitative similarities (see SI part 11). Partial surface coverages (commonly reported in thermal catalysis microkinetic modeling studies) can be obtained by dividing the surface densities values presented below with the active site density, ξ_tot_. Figure 4 presents the most important surface (panels a and b) and gas phase (panel c) reaction rates for the plasma-catalysis case at 500 K, with all values scaled per unit volume to facilitate comparison.

Surface densities for catalysis-only and plasma-catalysis cases are initialized at a value ξ_*i*_ = 1 × 10^5^ m^–2^ for all species to approximate the state of an empty catalyst surface (Figure 3). The high density of methane in the gas phase leads to the almost instantaneous rise of CH_3_* and H* surface densities to 2 × 10^15^ m^–2^ (partial coverage of 0.011%) at the onset of the simulation. For both reactor cases and for approximately 1 ms, the evolution of these primary surface species is identical, rising concurrently up to a value of 2.55 × 10^18^ m^–2^ (partial coverage of 13%). During this period, the dissociative adsorption of methane is dominant, while its associative desorption gradually rises in rate as the coverage of CH_3_* and H* increases (Figure 4a). The duration of this initial stage, and to a smaller degree the values that the surface densities of CH_3_* and H* approach, is affected by temperature. At 300 K, approximately 0.2 s is required for CH_3_* and H* to stabilize at 6 × 10^18^ m^–2^, whereas at 600 K, only 0.1 ms is needed to stabilize at 2 × 10^18^ m^–2^ (see SI part 11).

In the catalysis-only case, the surface is primarily covered by CH_3_* and H*, the densities of which remain 4 orders of magnitude higher than the next two most abundant species, CH_2_* and C_2_H_5_* ( Figure 3a). CH_2_* originates from the dehydrogenation of CH_3_*, while C_2_H_5_* is from the coupling of CH_3_* and CH_2_* species (Section 3.4). C_2_H_4_* also forms via coupling of CH_x_* (more details in Section 3.4.1), while species such as CH* and C_2_H_3_* appear in later simulation stages due to secondary dehydrogenations of other surface species.

Substantial differences are observed in surface species coverage trends during plasma-catalysis (Figure 3b). After CH_3_* and H* reach a plateau at approximately 1 ms, their surface densities start to diverge, with that of H* increasing further and that of CH_3_* decreasing gradually. The accumulation of H* on the studied time scales, and after 1 ms, is the result of the adsorption/desorption cycle of molecular H_2_ being unbalanced (Figure 4b) on account of complex plasma–catalyst interactions. The rapid dehydrogenation of ethane into ethylene in the gas phase via electron collisions (Figure 4c) enhances the catalytic production of ethane via the formation of C_2_H_4_* (see Section 3.4). In turn, this promotes the production of gas phase H_2_ and surface H*. This cycle also explains the fast and dominant production of ethane versus other C_2_ species discussed in the previous section. The progressive saturation of the catalyst surface with H* results in the drastic decrease after 0.018 s of the net adsorption of methane (Figure 4a) and the catalyst overall having a negative impact on the conversion of methane. This trend is reinforced by Eley–Rideal processes (CH_3_ + CH_x_* → CH_4_ + CH_*x*–1_*) that enhance the production of methane and contribute to the dehydrogenation of surface CH_x_* species. Eventually, the surface stabilizes to a state where CH_3_* production (and methane conversion) is sustained by the dissociative adsorption of CH_4_(ν1, 3) and CH_4_(ν2, 4) and the direct adsorption of CH_3_ (formed via CH_4_* (7.9 eV) self-dissociation within the plasma). In parallel, the production of ethane, and consequently its dehydrogenation by electron collisions, and the net adsorption of H_2_ (Figure 4b and c) decrease.

The formation of C_2_H_5_* and C_2_H_4_* follows the formation of CH_3_*, with their densities declining concurrently (Section 3.4). C_2_H_2_* is mostly produced from the adsorption of C_2_H_2_ at the conditions considered in these simulations. The formation of C_2_H* and C_2_H_3_* ensue from the formation of C_2_H_2_*. Atomic carbon C*, despite its strong binding with Ni(111), maintains very low densities at all stages of the simulation.

### 3.3 Turnover Frequencies (TOFs)

The catalysis-only and plasma-catalysis cases are further analyzed in terms of the turnover frequency (s^–1^) of methane (moles consumed) and ethane (moles produced) according to
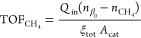
9
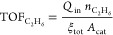
10where ξ_tot_ (sites·m^–2^) is the site density and *A*_cat_ (m^2^) is the total surface of the catalyst. It is assumed that there is no gas expansion in the reactor, so the reactor inlet and outlet volumetric flows are equal.

**Figure 5:**
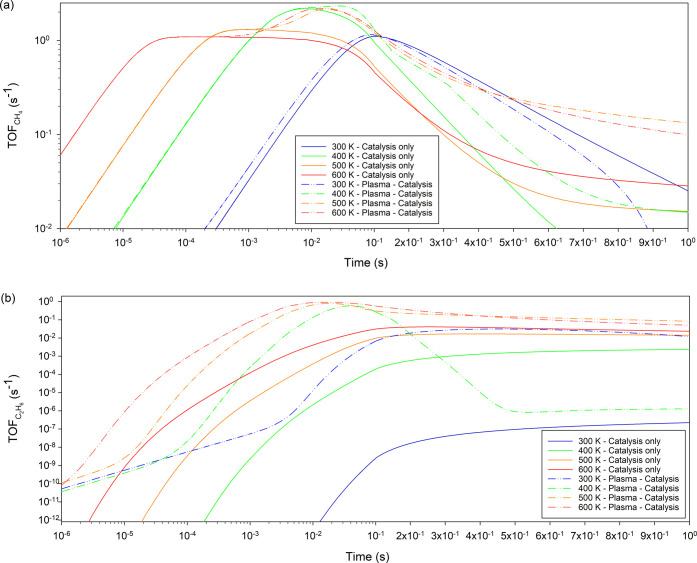
TOF (s^–1^) over time (s) (for times below 0.1 s, the axis is logarithmic, and linear afterward) for the catalytic scenarios and different temperatures studied for (a) methane and (b) ethane.

TOF_CH_4__ profiles naturally mirror those of methane density discussed in Section 3.1. For both reactor cases, the initial increase in TOF_CH_4__ occurs earlier at higher temperatures (Figure 5a). For catalysis-only, the lower peak value at 300 K indicates the slower adsorption at this temperature, while the higher peak value at 400 K followed by a sharper decrease is due to the faster adsorption and consequent saturation of the catalyst. At 500 and 600 K, TOF_CH_4__ reaches a plateau when the adsorption and desorption of CH_4_ equilibrate. The decrease in TOF_CH_4__ visible for all temperatures is due to the negative effects of the catalyst on the conversion of methane discussed previously. As the simulations approach steady state at approximately 1 s, TOF_CH_4__ at 600 K is higher than at 500 K due to a faster adsorption/desorption cycle. At 300 and 400 K, TOF_CH_4__ profiles continue decreasing, indicative of the eventual catalyst activity loss discussed in Section 3.1.

Plasma-catalysis TOF_CH_4__ profiles follow initially those of catalysis-only. At 300 K, minor differences observed are due to the higher adsorption rates induced by vibrationally excited species and the activation of methane in the gas phase. At higher temperatures, the synergistic effects observed on methane conversion (see Section 3.1) are again visible, with TOF_CH_4__ peak values being larger by a factor of ~ ∼2 at 500 and 600 K in comparison to the respective catalysis-only case. A slower decrease in the later stages of the simulations compared to catalysis-only is also visible, indicative of the beneficial effect of methane activation via plasma processes, despite the still present negative effects of the catalyst presence.

TOF_C_2_H_6__ profiles differ over time (Figure 5b), with temperature again being determining. In the catalysis-only case, values obtained at 500 and 600 K are approximately 1 and 5 orders of magnitude higher than those at 400 and 300 K, respectively. Plasma-catalysis cases demonstrate a significant increase in the peak TOF_C_2_H_6__ values compared to the equivalent catalysis-only cases for all temperatures considered, confirming the existence of synergism. The decrease in TOF_C_2_H_6__ in later stages of the simulation is more pronounced at 400 K, as a consequence of the unbalanced adsorption/desorption cycles, which saturate the catalyst faster than in the other cases.

Table 5 summarizes the TOF_CH_4__ values obtained in this work at 500 and 600 K for the two catalysis cases studied. The data obtained at the end of the simulations (for a time of 1 s in Figure 5) are considered to be steady-state values for these temperatures, while peak values from Figure 5 are also provided for comparison. In Engelmann et al.,^25^ one of the very few works that have studied the plasma-catalytic nonoxidative methane via microkinetics, rates at 0% conversion were provided for a variety of transition metals. For Ni(111), at 500 K and a vibrational temperature of 1500 K, initial TOF_CH_4__ values approximately equal to 1 s^–1^ were reported when reactive plasma species were considered, which are in overall good agreement with the present peak values. Nonetheless, the current work resolves further the temporal evolution of TOF_CH_4__, revealing that the high initial (or peak) rates are not maintained, with the catalyst effectively obstructing plasma-driven pathways. In all cases, the proximity of results reported in the two works, within their methodological differences, suggests that UBI-QEP, despite its semiempirical nature, can be used to estimate surface energetics, particularly in cases where DFT data for obtaining scaling relationships are not readily available. Experimental comparison is more challenging, as methane conversion is typically studied at higher temperatures than those evaluated in the present work. Indicatively, Table 5 presents TOF_CH_4__ values that were reported in methane decomposition experimental works over Ni catalysts. For methane decomposition at 873 K over a Ni/Al_2_O_3_ catalyst, TOF_CH_4__ values of 0.75 and 0.18 s^–1^ after 0.5 and 3 h of reaction time were reported,^58^ while over Ni/MgO at the same temperature, values from almost 4 to 1.69 s^–1^ were measured, the decline in rate observed being within only 2 min. Clearly, experimental results are impacted by carbon deposition, an aspect not accounted for in the present simulations. Moreover, catalyst structural features like the support material, the metal particle size, and dispersion further impact the experimentally measured rates. This was exemplified in the work of Xu et al.,^59^ where variation of Ni particle size was seen to be the main controlling parameter for methane decomposition rates. At temperatures closer to those modeled in the current work, Kechagiopoulos et al. reported TOF_CH_4__ values for methane decomposition from 0.1 to 0.32 s^–1^. In all cases, an order of magnitude agreement with catalysis-only results is observed across a range of experimental works. In combination with the multiple reports successfully having used UBI-QEP^60−63^ or Polanyi relationships^64,65^ together with chemisorption energies as catalyst descriptors for the microkinetic modeling of high-temperature heterogeneously catalyzed methane conversion over various metals, an adequate depiction of the purely catalytic reactivity is suggested. Nonetheless, more research is warranted toward a detailed validation of plasma-catalysis microkinetic models.

**Table 5:** TOF_CH_4__ Values Obtained via Plasma-Catalysis Microkinetic Simulations and Experimental Results Obtained with Ni on Various Supports for Methane Decomposition

source	type of work	temperature	TOF_(CH_4_)_ (s^–1^)	
Engelmann et al.^25^	plasma-catalysis microkinetic modeling on Ni(111)	*T*_0_ = 500 K	≈1 (0% conversion)	
		*T*_vib_ = 1500 K		
Ray et al.^58^	decomposition over Ni/Al_2_O_3_	*T*_0_ = 873 K	0.75 (after 0.5 h)	
			0.18 (after 3 h)	
Wei and Iglesia^66^	decomposition over Ni/MgO	*T*_0_ = 873 K	3.87 (initial)	
			1.69 (after 2 min)	
Xu et al.^59^	decomposition over Ni on various supports	*T*_0_ = 923 K	0.52–2.89 across 9–19 nm Ni particle sizes	
Kechagiopoulos et al.^63^	decomposition over Ni/La_2_O_3_ – CeO_2_ – ZrO_2_	*T*_0_ = 673 K	≈0.1	
		*T*_0_ = 773 K	≈0.32	
this work	catalysis microkinetic modeling on Ni(111)	*T*_0_ = 500 K	1.30 (peak)	0.015 (final)
		*T*_0_ = 600 K	1.09 (peak)	0.028 (final)
	plasma-catalysis microkinetic modeling on Ni(111)	*T*_0_ = 500 K	2.18 (peak)	0.129 (final)
		*T*_0_ = 600 K	1.15 (peak)	0.949 (final)

### 3.4 Reaction Pathway Analysis (RPA)

A reaction pathway analysis is carried out to elucidate the importance of production and consumption pathways in the transformation of species. Rates for the different reactor cases are integrated over a time of 2 ms (*t*_integ_) and normalized with their characteristic dimension, namely, the total volume available (*V*_reac_ or *V*_void_) for gas rates (*r*_*i*,*g*_) and the total area of the catalyst, *A*_cat_, for surface rates (*r*_*i*,*s*_)
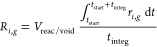
11
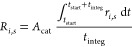
12In the above, *R*_*i*_ indicate the integrated normalized rates in particles·s^–1^, which provide the number of molecules, radicals, etc. that are produced/consumed during 2 ms.

#### 3.4.1 Carbon Transformations

Carbon transformations at 500 K are considered at the time when the net rate of CH_4_ consumption is at its peak (Section 3.1), with rate integration (eqs 11 and 12) taking place for 2 ms following the peak time. For plasma-only cases, where CH_4_ consumption profiles do not peak, integration of rates takes place toward the end of the simulation when steady state has been reached. For comparison, a similar analysis is performed at a later stage of plasma-catalysis simulations when activity has decreased and stabilized.

**Figure 6:**
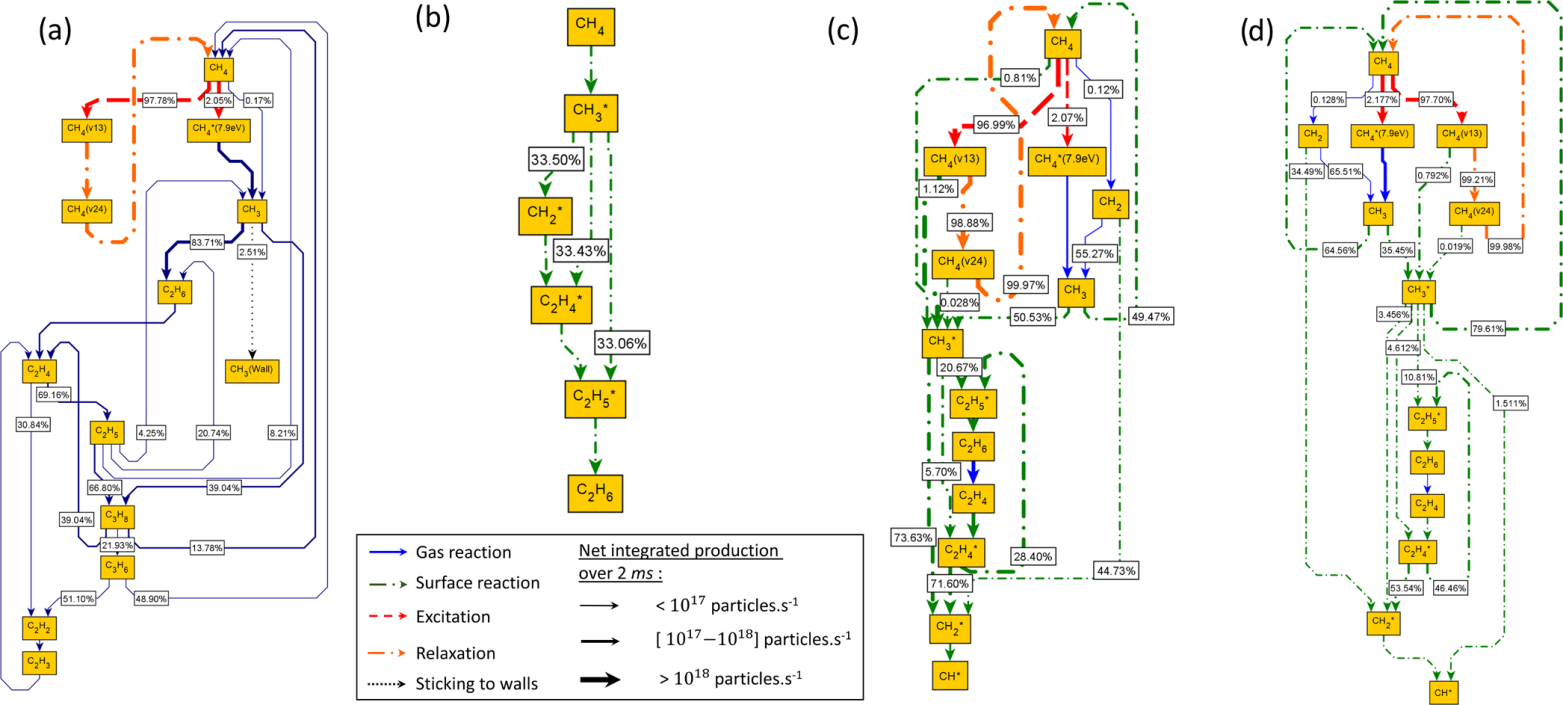
Reaction pathway analysis of carbon at 500 K and at maximum CH_4_ consumption for (a) plasma-only, (b) catalysis-only, and (c) plasma-catalysis cases, and for stabilized performance for (d) the plasma-catalysis case.

Figure 6a presents the reaction pathway analysis for the plasma-only case at simulation time *t*_start_ = 1.45 s. Results follow those previously elaborated,^44^ with the cycle of excitation/de-excitation of vibrationally excited states of methane rates being 2 orders of magnitude faster than the conversion rates of these states into CH_3_. The creation of methyl radicals mostly takes place via the dissociation of the electronically excited state CH_4_* (7.9 eV). The primary product is ethane, which further dehydrogenates to acetylene via electron collisions. The sticking of CH_3_ radicals on the walls of the reactor is limited, accounting for 2.51% of its total consumption.

Catalysis-only reaction pathways at peak simulation time *t*_start_ = 0.39 *s* are much simpler (Figure 6b). Due to the absence of electron collisions and the temperature of 500 K being too low to thermally activate methane, gas phase activity is negligible. Activation of methane takes place only at the surface of the catalyst via its dissociative adsorption to CH_3_* and H*. CH_3_* is equally consumed by coupling and H-transfer pathways
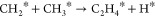

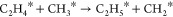
Both processes enable the formation of C_2_H_5_*, which ultimately desorbs associatively as ethane. Eley–Rideal processes have a minor contribution to conversion pathways as radicals are scarce at the considered temperature.^56^ The most populated radical is CH_3_, whose average density of only 1.0 × 10^7^ m^–3^ is orders of magnitude lower that those observed during plasma-only cases (1.0 × 10^20^ m^–3^) and plasma-catalysis (1.0 × 10^18^ m^–3^) (H, CH_3_, and C_2_H_5_ density profiles available in SI part 9).

Plasma-catalysis reaction pathways at peak simulation time *t*_start_ = 0.018 s share similarities to those of the catalysis-only case but are significantly more complex (Figure 6c). Multiple methane activation and excitation mechanisms are present in the plasma phase, enabling also new pathways at the surface of the catalyst. As with the plasma-only case, the cycle of vibrational excitation of methane and its de-excitation via VV and VT processes (integrated rates of ≈5 × 10^20^ particles·s^–1^) is much faster than any other process (most have integrated rates in the range of ≈5 × 10^18^ particles·s^–1^). The electronical excitation of methane accounts for the same conversion percentage as in the plasma-only case (2.07%), with CH_4_* (7.9 eV) exclusively self-dissociating toward CH_3_. Methyl radicals either directly adsorb on the catalyst (CH_3_ + * → CH_3_*) at a high conversion contribution of 50.53% or convert back to ground-state methane via the following Eley–Rideal reactions at respective contributions of 56.0, 39, 7, and 3.4%
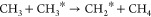

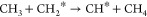

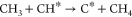


Adsorption and desorption rates of CH_4_ are in the same order of magnitude as those of VT-VV processes (≈5 × 10^20^ particles·s^–1^); however, being quasi-equilibrated (Section 3.2), the net rate of CH_4_ adsorption (5 × 10^18^ particles·s^–1^) accounts for only 0.81% of its total consumption. Noteworthy is the enhanced adsorption of CH_4_(ν2, 4) and CH_4_(ν1, 3), which provides new channels toward the formation of CH_3_*. In line with the results discussed in Section 3.2 and in the literature,^25,67^ a higher fraction of CH_4_(ν1, 3) (1.12%) adsorbs on the catalyst in comparison to the other methane states considered (0.81% for CH_4_ and 0.028% for CH_4_(ν2, 4)).

CH_3_* is the primary species enabling interaction between plasma and the catalyst (Section 3.2). Also, 73.63% of CH_3_* dehydrogenates to CH_2_* according to the Eley–Rideal reactions presented earlier and surface H-transfer (C_2_H_4_* + CH_3_* → C_2_H_5_* + CH_2_*), leading to the shift in surface coverages of these species discussed in Section 3.2 and explaining the net dehydrogenation of CH_3_* to CH_2_* visible in Figure 6c. C_2_H_5_* desorbs as C_2_H_6_, the latter dehydrogenating in the gas phase via electron collisions (e + C_2_H_6_ → e + C_2_H_4_ + H_2_). Ethylene interacts strongly with the catalyst, with nearly 100% of its consumption being due to its adsorption (C_2_H_4_ + * → C_2_H_4_*). Again, 78.10% of the formation of C_2_H_4_* is due to adsorption, the rest originating from CH_x_* coupling
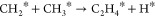
The presence of C_2_H_4_* enables two important surface pathways that are responsible for 79.53 and 20.47% of its consumption
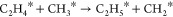

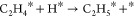
These processes enhance the production of C_2_H_5_* and, consequently, that of ethane in the gas phase versus other C_2_ products. The plasma-catalysis process is characterized by complex dynamic interactions, whereby the production of ethane by the catalyst induces the production of ethylene in the gas phase, which in turn contributes to the consumption of CH_3_* toward the formation of C_2_H_5_* and, ultimately, ethane production in the gas phase.

CH_2_* further dehydrogenates to CH* mostly via Eley–Rideal processes CH_3_ + CH_2_* → CH* + CH_4_ and CH_2_ + CH_2_* → CH* + CH_3_ at respective contributions of 91.50 and 4.54%, while the C* formation is minor due to its effective hydrogenation via the process CH_3_ * + C* → CH_2_ * + CH*. Finally, acetylene produced in the gas phase mostly adsorbs on the catalyst. C_2_H_2_* is the only source of C_2_H* and C_2_H_3_*, the latter species converting solely to C_2_H_4_* (pathways omitted in Figure 6c due to their low rate of 5 × 10^13^ particles·s^–1^).

A similar analysis is performed at a later stage of the plasma-catalysis simulation (simulation time *t*_start_ = 1.05 s), when the catalyst was observed to have a detrimental effect on the conversion of methane (Section 3.1). The results presented in Figure 6d resemble the pathways discussed for the peak performance (Figure 6c), exhibiting however two notable differences. Most importantly, the net production between CH_4_ and CH_3_* is in the direction toward methane, indicative of its desorption at this stage being faster than its adsorption. Additionally, the net rate between C_2_H_6_ and C_2_H_4_ in the gas phase is approximately ten times lower than that at peak performance, as at this stage of the simulation, the hydrogen produced from ethane’s dehydrogenation is responsible for the overall lower activity.

#### 3.4.2 Effect of Temperature on the Reaction Pathways of CH_3_* and H*

**Figure 7:**
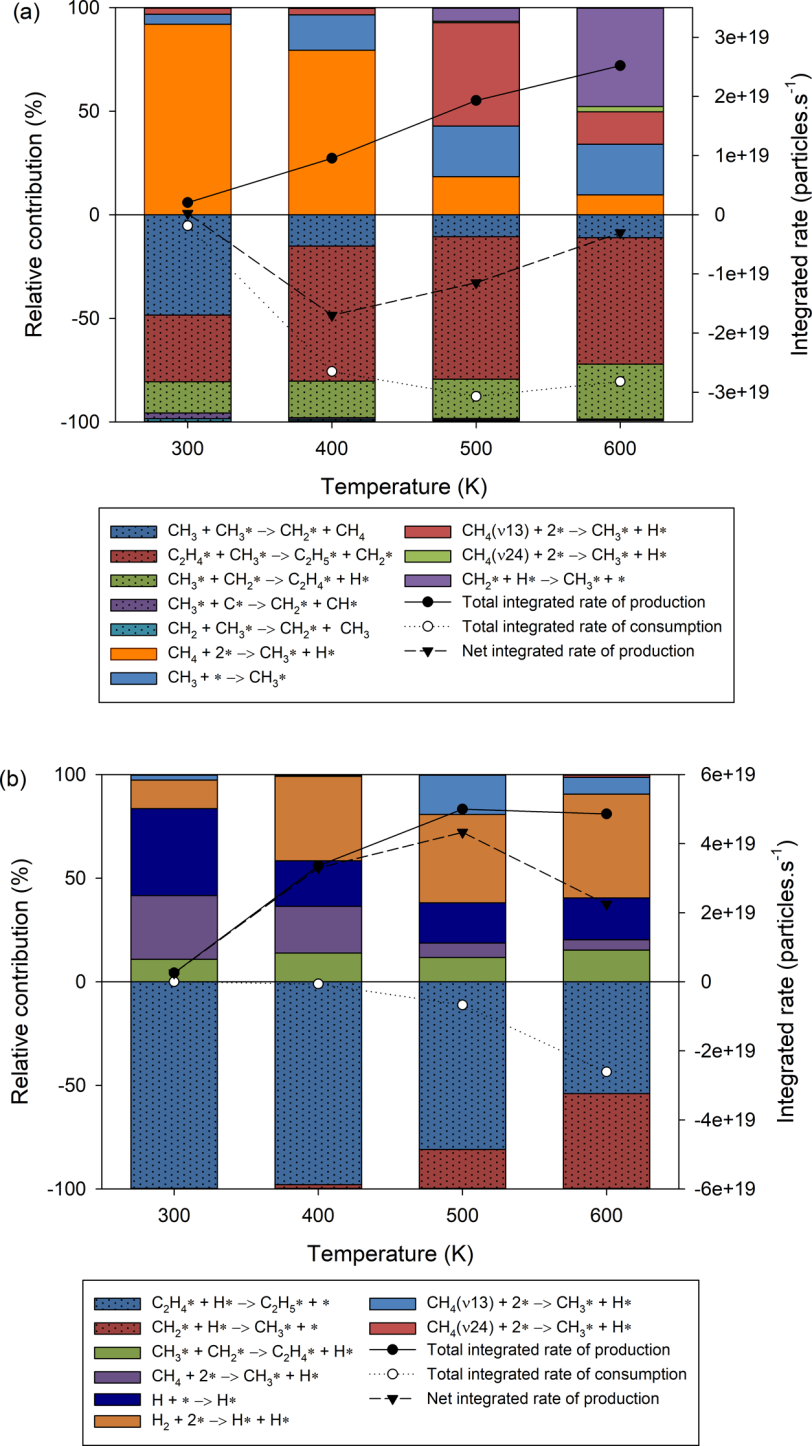
Reaction pathway analysis of (a) CH_3_* and (b) H*. Relative contribution (%) and total integrated rates (particles·s^–1^) over temperature (K).

A contribution analysis is further performed for the plasma-catalysis case with focus on CH_3_* and H* species to investigate the impact of gas temperature. As in the previous section, normalized rates are integrated for 2 ms starting at the peak time in CH_4_ consumption. Net rates are calculated for reversible processes and are written in the dominant direction. Results are presented in Figure 7, while in the SI (part 12), a similar analysis is provided for C_2_H_5_* and C_2_H_4_* species whose pathways showed much less variation across temperature.

At 300 K, CH_3_* accumulates on the catalyst (confirmed by its integrated net rate of formation being marginally positive). Conversely, at all other temperatures, CH_3_* acts as a reactant, as indicated by its negative net rate of formation (Figure 7a). Consumption of CH_3_* is the most pronounced at 400 K due to the slow desorption of methane, while at 500 and 600 K, the main CH_3_* consumption processes involve surface coupling and hydrogen transfers toward C_2_H_5_* and C_2_H_4_* formation. The relative contribution of the Eley–Rideal process CH_3_ + CH_3_* → CH_2_* + CH_4_ decreases with temperature. The contribution of the dissociative adsorption of ground-state methane in the production of CH_3_* decreases drastically above 400 K as the reverse process accelerates at 500 and 600 K. At 400 K and higher, the dissociative adsorption of vibrationally excited states of methane is dominant. With net rates of 6.45 × 10^16^, 2.99 × 10^16^, 9.60 × 10^18^, and 3.93 × 10^18^ particles·s^–1^ at 300, 400, 500, and 600 K, respectively, the dissociative adsorption of CH_4_(ν1, 3) is seen to maximize at 500 K. At this temperature, a balance between catalyst activity and excited species populations is achieved, with CH_4_(ν1, 3) adsorption rates being higher than at 300 and 400 K, but rates of VV processes being lower than at 600 K (Section 3.1). These positive effects manage to counterbalance the lower density of CH_4_(ν1, 3), which overall has a higher importance in CH_3_* production than CH_4_(ν2, 4) for all considered temperatures. These findings are primarily an outcome of the higher internal energy of the CH_4_(ν1, 3) state versus that of CH_4_(ν2, 4); however, qualitatively, they compare well with the works of Juurlink et al.^67^ and Chen et al.^68^ on Ni(111), who found the stretching modes to be much more efficient in promoting methane adsorption than the bending modes.

The accumulation of H*, discussed throughout this work, is confirmed by its positive integrated net rate of formation (Figure 7b). At 300 and 400 K, the consumption of H* is effectively zero. The production of H* via the adsorption of the H radical is pronounced at 300 K, whereas at higher temperatures, the dissociative adsorption of H_2_ increases in importance as an outcome of the dehydrogenation of ethane in the gas phase, which enhances also the H_2_ density. Reaction pathway analysis results for hydrogen presented in the SI (part 13) indicate that the dissociative adsorption of H_2_ is even faster than its vibrational excitation. Finally, similar trends to those observed for CH_3_* production by the adsorption of CH_4_(ν1, 3) and CH_4_ are visible also for the formation of H*, although to a less pronounced effect.

### 3.5 Energy Efficiency

The energy efficiency *E*_eff_(*T*_0_) for the plasma-catalysis and plasma-only cases is compared at different temperatures studied. The energy efficiency is defined as the ratio of the reaction enthalpy of the overall transformation of methane to the energy cost of the plasma
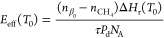
13with *N*_A_ being the Avogadro number. For the estimation of Δ*H*_r_(*T*_0_), the selectivities of products, *S*_C_*x*_H_*y*_,*i*_, and their corresponding reaction enthalpies from methane at the respective temperature, Δ*H*_*r*,*i*_(*T*_0_), are considered (see SI part 14 for details)
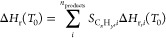
14As with the reaction pathway analysis, the energy efficiency is calculated at the peak performance time of the simulations. Moreover, the impact of the specific surface area of the catalyst, *a*_cat_, is investigated by studying values up to 25 times higher than the base case (*a*_cat_ = 1050 m^2^·g^–1^). Due to the very low activity observed at 300 K, this temperature is omitted from the analysis. Results are compared also to a theoretical maximum, which corresponds to the energy efficiency obtained for the complete conversion of methane to ethane at the power density of plasma-catalytic runs, namely, when *n*_CH_4__ = 0 m^–3^, *S*_C_2_H_6__ = 1 (−), and *P*_d_ = 9.1 × 10^7^ W·m^–3^ (see also Section 2.2).

The theoretical maximum energy efficiency is found to be approximately 14%, with minor variation among the different temperatures studied. This corresponds to an optimal energy cost in this DBD reactor of 5.79 MJ·mol_CH_4__^–1^, which compares well with the values reported by SriBala et al.^69^ in a glow electrical discharge ranging from 5 to 5.5 MJ·mol_CH_4__^–1^. These values are directly derived from methane conversion, which mainly depends on the molar flow of methane and the power density in the reactor.^42^ The low energy efficiency even at the optimal case is a frequently reported issue with atmospheric pressure DBD reactors, which are subject to substantial energy losses due to the relaxation of excited species. Atmospheric pressure thermal discharges such as gliding arcs or sparks achieve efficiencies from 40 to 50% as they benefit from both thermal and plasma activation of methane. Microwave plasmas operating at low pressure reach efficiency values of approximately 20%,^41^ also higher than DBD.

**Figure 8:**
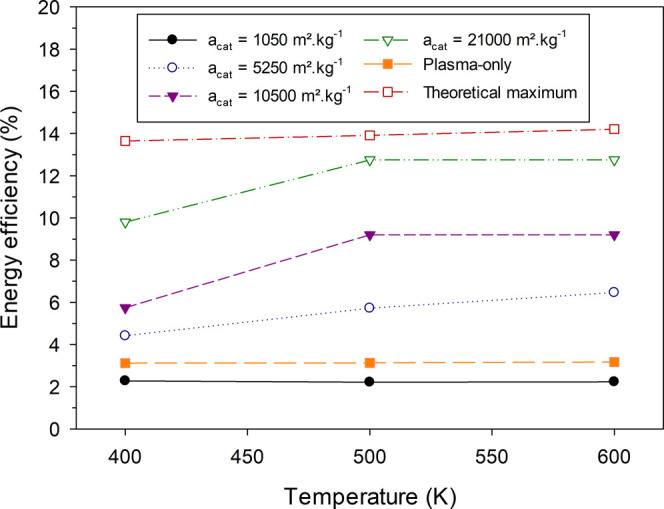
Energy efficiency (%) over gas temperature (K) for the plasma-only case and various specific surface areas of the catalyst, *a*_cat_, in the plasma-catalysis case.

The energy efficiency for the plasma-only case is approximately 3% (Figure 8), consistent with various works having reported values below 10%.^18,41,43,70^ As mentioned, this poor energy efficiency is mainly due to the high proportion of energy consumed in the vibrational excitation of methane being lost through relaxation processes.^44,71^

The base plasma-catalysis case (*a*_cat_ = 1050 m^2^·g^–1^) has an even lower efficiency compared to the plasma-only case at about 2% on account of two effects. First, Δ*H*_r_(*T*_0_) is about 50% lower for plasma-catalysis, as the selectivity toward ethane is 100%, while in plasma-only cases, all C_2_ species have approximately the same selectivity. Second, in plasma-catalysis, the power density is higher compared to plasma-only. However, as can be seen in Figure 8, the energy efficiency increases substantially at higher specific surface areas, reaching close to the theoretical maximum for a value of 21 000 m^2^·g^–1^. These results agree well with the work of Kasinathan et al.,^72^ whose energy efficiency was estimated to be 10.8%,^41^ and the work of Taheraslani and Gardeniers,^18^ who reported energy efficiencies from 8 to 11% in the presence of a γ-alumina-supported Pd catalyst.

## 4 Conclusions

The nonoxidative coupling of methane over Ni(111) was studied via a dynamic 0D plasma-catalytic model, comprising detailed plasma and surface reaction networks, explicitly describing the interactions of plasma and surface species. A gas bulk temperature from 300 to 600 K was considered with equivalent plasma-only and catalysis-only cases also simulated to discern the plasma and catalyst contributions. Results showed that significant synergistic effects could only be observed at 500 K and above, where a fast turnover of species on the catalyst was achieved. In good agreement with the experimental literature, Ni(111) was found to be selective almost exclusively toward ethane, with only traces of ethylene also being produced. However, molecular hydrogen originating from the dehydrogenation of ethane in the plasma was seen to progressively saturate the catalyst surface, which reduced the performance of the plasma-catalysis system by promoting hydrogenation of CH_3_* back to methane.

The model further allowed us to observe the effect of reactive species from the plasma, such as vibrationally excited methane and radicals, on the surface species and reactions. The most notable contribution was that of CH_4_(ν1, 3) at 500 K, a temperature where the species was found to be efficiently adsorbing of the catalyst, with VV processes not consuming it excessively in the plasma. The direct adsorption of CH_3_ from the gas phase was significant; however, the same radical was also observed to accelerate the formation of CH_4_ via Eley–Rideal reactions on CH_x_* surface species.

Finally, the energy efficiency of the process was found to be highly sensitive to the area-to-volume ratio of the catalyst. Plasma-catalysis does not necessarily lead to better efficiency compared to plasma alone, stressing the need for operating conditions’ optimization. The model presented in this work can be employed to design experiments, while using the heats of chemisorption of surface species as catalyst descriptors allows the investigation of other metals in a straightforward manner.

## Supporting Information Available

Catalytic bed characteristics; kinetic model parameters’ calculation; list of reactions in the model; density profiles of selected species in linear scale; density profiles of the main radicals; product selectivities and methane conversion tables; surface density profiles for all temperatures; reaction pathway analyses for C_2_H_4_*, C_2_H_5_*, and H_2_; and reaction enthalpies considered in the energy efficiency calculations (PDF)
