# Supplementary material for: Plasma-Catalysis of Nonoxidative Methane Coupling: A Dynamic Investigation of Plasma and Surface Microkinetics over Ni(111)
Source: J Phys Chem C Nanomater Interfaces. 2022 Nov 17;126(47):19987–20003. doi: 10.1021/acs.jpcc.2c03503 (PMC9720725; doi:10.1021/acs.jpcc.2c03503)
Supplement: Supplementary file 1 — jp2c03503_si_001.pdf [file jp2c03503_si_001.pdf]

Supporting Information

Plasma-Catalysis of Non-Oxidative Methane Coupling: A Dynamic Investigation of Plasma and Surface Microkinetics over Ni(111)

Pierre-André Maitre, Matthew S. Bieniek, and Panagiotis N. Kechagiopoulos*

Chemical Processes & Materials Group, School of Engineering, University of Aberdeen, Aberdeen, AB24 3UE, UK

E-mail: p.kechagiopoulos@abdn.ac.uk

Contents

1 Catalytic bed characteristics **S2**

2 Debye length and plasma processes inside the catalyst pores **S3**

3 Thermodynamic consistency of surface reaction network **S5**

4 Kinetic model parameters calculation **S8**

Pre-exponential factors: S9

Activation energies and reaction enthalpies: S9

Reactivity of vibrationally excited states: S11

5 Adsorption processes **S12**

5.1 Dissociative processes S12

5.2 Molecular adsorptions S12

6 Surface reactions S13

7 Eley-Rideal processes S14

8 Density profiles of CH_4 and C_2 species in linear scale S15

9 Density profiles of main radicals S15

10 Product selectivities and methane conversion S16

11 Temperature effect on surface densities S18

12 Effect of temperature on the reaction pathways of C_2H_4* and C_2H_5* S20

13 Hydrogen transformations in plasma-catalysis S22

14 Reaction enthalpies considered in the energy efficiency calculations S23

References S24

1 Catalytic bed characteristics

The total volume of the reactor, V_{reac}, and the surface of the walls, A_{walls}, are obtained directly from the geometry of the DBD reactor. The volume of the catalytic bed, V_{bed}, corresponds to the total volume occupied by the pellets, while the void left between the pellets is $V_{void-bed}$. The total void inside the pellets, $V_{void-pellets}$, has the same volume as that occupied by the solid, V_{solid}, as the porosity of the pellets ϕ_{pellet}, is equal to 0.5. The total mass and surface area of the catalyst in the reactor are M_{cat} and A_{cat}, respectively. The interstitial distance between pellets, $d_{inter-pellet}$, represents the average distance between two catalyst pellets, while the average internal pore diameter within the pellets is $d_{intra-pellet}$. Formulas used and respective values estimated for these characteristics are provided in the table below.

Table S1: Catalytic bed characteristics calculated based on the considered catalyst and geometrical arrangement.

Characteristic	Calculation	Value
$V_{reac}\,(m^3)$	$\pi\, L\,(R_d^2 - R_g^2)$	7.54×10^{-7}
$A_{walls}\,(m^2)$	$2\,\pi\, L\,(R_d + R_g)$	7.54×10^{-4}
$V_{bed}\,(m^3)$	$V_{reac}(1-\phi_{bed})$	4.15×10^{-7}
$V_{void-bed}\,(m^3)$	$V_{reac}\,\phi_{bed}$	3.39×10^{-7}
$V_{void-pellet}\,(m^3)$	$V_{bed}\,\phi_{pellet}$	2.07×10^{-7}
$V_{solid}\,(m^3)$	$V_{bed}\,(1-\phi_{pellet})$	2.07×10^{-7}
$V_{void-total}\,(m^3)$	$V_{void-pellets} + V_{void-bed}$	5.46×10^{-7}
$M_{cat}(g)$	$\rho_{cat}\,V_{solid}$	0.477
$A_{cat}\,(m^2)$	$a_{cat}\,M_{cat}$	0.501
$d_{inter-pellet}(mm)$	$\frac{4}{3}d_{pellet}(\frac{\phi_{bed}}{1-\phi_{bed}})$	2.182×10^{-1}
$d_{intra-pellet}(mm)$	$\frac{4\phi_{pellet}}{\rho_{cat}a_{cat}}$	8.282×10^{-4}

2 Debye length and plasma processes inside the catalyst pores

The vast majority of the active sites of a catalyst are located within the pores of its pellets. Ensuring gas phase species have access to these sites is critical for heterogeneous catalysis[1]. Plasma contains highly reactive species, such as radicals and excited states, which can also react on the catalyst active sites, leading to synergistic effects where plasma-catalysis performs better than the equivalent plasma-only and catalysis-only cases[2–5]. However, a commonly raised issue of plasma-catalysis[6–9] is ensuring the plasma is as homogeneous as possible within the catalytic bed, in order to maximize the contact between plasma species and catalyst active sites. For the case of DBD discharges, which, by nature, operate in a non-homogeneous filamentary regime populated by streamers, it needs to be determined at which conditions the streamers are able

to penetrate the pores of the catalyst. Zhang and Bogaerts[10,11], have estimated, using Particle In Cell and Monte Carlo Collision modelling, that the Debye length is an important criterion in assessing this, as streamers can only penetrate pores with a diameter larger than this length. The Debye length, λ_D, in low temperature plasma can be calculated according to:

$$\lambda_D = \sqrt{\frac{\varepsilon_0\, k_b\, T_e}{n_e\, e^2}}$$

with ε_0 being the void permittivity ($8.8542 \times 10^{-12}\, F.m^{-1}$), k_b the Boltzmann's constant ($1.3806 \times 10^{-23}\, J.K^{-1}$), T_e the electron temperature (K), n_e the electron density (m^{-3}) and e the elementary charge ($1.602 \times 10^{-19}\, C$).

The Debye length is estimated, for all the different gas temperatures considered, based on the steady-state results of plasma-only cases, and is compared with values from literature (Tables S2 and S3). From Table S2, it is clear that the homogeneous nature of the simulation decreases greatly the population of electrons, n_e, as the power input density is 2 to 3 orders of magnitude lower than what is generally considered in pulse models[12–14]. Consequently, in this homogeneous model, the electron density encountered is up to 4 orders of magnitude lower than typical populations in streamers (Table S3), inevitably resulting in a Debye length that is about 1 to 2 orders of magnitude lower than that of typical DBD streamers.

Table S2: Electron properties and Debye lengths obtained with the plasma-only 0D homogeneous model at steady-state for the different gas temperatures studied.

**Gas temperature (K)**	$n_e(m^{-3})$	$T_e(K)$	λ_D (mm)
300 - 600	4.24×10^{15}	2.96×10^4	0.182

Table S3: Typical literature values of streamers characteristics.

$n_e(m^{-3})$	$T_e(K)$	λ_D (mm)	**Source**
1×10^{17}	2.32×10^4	0.0332	[15] (averaged data)
3×10^{19}	3.48×10^4	0.00235	[12] (peak data)
1×10^{19}	4.20×10^4	0.00447	[16] (peak data)

Debye lengths calculated for all the different gas temperatures of the plasma-only case are similar in magnitude (Table S2). For the plasma-catalysis cases, they are expected to be

shorter at the equivalent gas temperature as a higher power density input P_d is considered compared to plasma-only cases. This means that for the simulated catalyst characteristics, plasma processes are present within the void of the catalytic bed but do not penetrate into the pores of the catalyst, as $d_{inter-pellet} > \lambda_D >> d_{intra-pellet}$.

3 Thermodynamic consistency of surface reaction network

When building microkinetic models of surface mechanisms, it is necessary to ensure that thermodynamic consistency is respected at both enthalpic and entropic levels[17,18]. Rates of forward and backward elementary reaction steps have to be calculated under thermodynamic constraints at both individual and reaction mechanism levels. The approach applied in the current work follows that presented in[19]. For every elementary reaction j considered in the model, the following relations hold between the respective forward and backward activation energies and pre-exponential factors[17]:

$$e^{\frac{\Delta S_{s,j}}{R}} = \frac{k^f_{0j}}{k^b_{0j}} \tag{1}$$

$$E^f_j - E^b_j = \Delta H_{s,j} \tag{2}$$

with $\Delta S_{s,j}$ and $\Delta H_{s,j}$ being the reaction entropy and enthalpy of a surface process, respectively, k^f_{0j} and k^b_{0j} being the forward and backward pre-exponential factors, E^f_j and E^b_j the forward and backward activation energy, and R the ideal gas constant ($8.314\, J.K.mol^{-1}$).

The use of thermodynamic relationships is also employed to reduce the number of adjustable parameters, via their correlation with the chosen catalyst descriptors (the heats of chemisorption of the surface species χ_i). The enthalpies and entropies of surface reactions are estimated via thermodynamic relationships with the analogous process in the gas-phase. The thermodynamic data of surface species are explicitly obtained through correlations.

The entropy of formation of a surface species, $S_{s,i}$, at a given temperature is obtained from the subtraction of the gaseous translational entropy, from the gas phase entropy of formation of

the corresponding gas species, $S_{g,i}$, according to:

$$S_{s,i} = S_{g,i} - S_{trans-3D,i} \tag{3}$$

with $S_{trans-3D,i}$ being the translational entropy of the gas phase species lost upon adsorption onto the surface.

Equation (3) implies that surface species are completely immobile on the catalyst and lose all three translational degrees of freedom upon adsorption[20,21]. The translational entropy is calculated as in[22]:

$$S_{trans-3D,i} = R\left[ln\left(\frac{(2\pi M_j k_b T_0)^{3/2}}{h^3}\right) + ln\left[\frac{k_b T_0}{p_0}\right] + \frac{5}{2}\right] \tag{4}$$

with h being Planck constant ($6.626 \times 10^{-34}\, m^2.kg.s^{-1}$).

Analogously, the enthalpy of formation of a surface species, $H_{s,i}$, at a given gas temperature is obtained by subtracting the heat of chemisorption, χ_i, of the equivalent gas phase species from its respective equivalent gas phase enthalpy of formation, $H_{g,i}$:

$$H_{s,i} = H_{g,i} - \chi_i \tag{5}$$

The standard enthalpy (H^0) and entropy (S^0) of formation of all gas phase species considered in the model are parametrised in the form of polynomial fits in function of T_0 following the NASA chemical equilibrium code format[23]:

$$\frac{S^0}{R} = \lambda_1 ln(T_0) + \lambda_2 T_0 + \frac{\lambda_3}{2}T_0^2 + \frac{\lambda_4}{3}T_0^3 + \frac{\lambda_5}{4}T_0^5 + \lambda_7$$

$$\frac{H^0}{RT_0} = \lambda_1 + \lambda_2\frac{T_0}{2} + \frac{\lambda_3}{3}T_0^2 + \frac{\lambda_4}{4}T_0^3 + \frac{\lambda_5}{5}T_0^4 + \frac{\lambda_6}{T_0}$$

The values of the coefficients λ_i for all species are provided in Table S4 with H^0 given in ($kJ.mol^{-1}$) and S^0 given in ($J.K^{-1}.mol^{-1}$).

Table S4: Thermodynamic parameters used for the calculation of H^0 and S^0.

Species	λ_1	λ_2	λ_3	λ_4	λ_5	λ_6	λ_7
H_2	3.298124310	$8.249441740\times10^{-04}$	$-8.143015290\times10^{-07}$	$-9.475434330\times10^{-11}$	$4.134872240\times10^{-13}$	$-1.012520870\times10^{+03}$	-3.294094090
H	2.500000000	0.000000000	0.000000000	0.000000000	0.000000000	$2.547162700\times10^{+04}$	$-4.601176080\times10^{-01}$
CH_4	$7.787415000\times10^{-01}$	$1.747668000\times10^{-02}$	$-2.783409000\times10^{-05}$	$3.049708000\times10^{-08}$	$-1.223931000\times10^{-11}$	$-9.825229000\times10^{+03}$	$1.372219000\times10^{+01}$
CH_3	2.430443000	$1.112410000\times10^{-02}$	$-1.680220000\times10^{-05}$	$1.621829000\times10^{-08}$	$-5.864953000\times10^{-12}$	$1.642378000\times10^{+04}$	6.789794000
CH_2	3.762237070	$1.159819080\times10^{-03}$	$2.489585430\times10^{-07}$	$8.800835620\times10^{-10}$	$-7.332435440\times10^{-13}$	$4.536790630\times10^{+04}$	1.712577580
CH	3.200202470	$2.072875620\times10^{-03}$	$-5.134431380\times10^{-06}$	$5.733890250\times10^{-09}$	$-1.955533180\times10^{-12}$	$7.045259380\times10^{+04}$	3.331587790
C	2.554239550	$-3.215377240\times10^{-04}$	$7.337922450\times10^{-07}$	$-7.322348890\times10^{-10}$	$2.665214460\times10^{-13}$	$8.544388320\times10^{+04}$	4.531308480
C_2H_6	1.462538720	$1.549466700\times10^{-02}$	$5.780507310\times10^{-06}$	$-1.257831880\times10^{-08}$	$4.586267130\times10^{-12}$	$-1.123917580\times10^{+04}$	$1.443229490\times10^{+01}$
C_2H_5	2.690701720	$8.719133210\times10^{-03}$	$4.419838660\times10^{-06}$	$9.338703140\times10^{-10}$	$-3.927773470\times10^{-12}$	$1.287040430\times10^{+04}$	$1.213819500\times10^{+01}$
C_2H_4	$-8.614879850\times10^{-01}$	$2.796162850\times10^{-02}$	$-3.388677210\times10^{-05}$	$2.785152200\times10^{-08}$	$-9.737878910\times10^{-12}$	$5.573045900\times10^{+03}$	$2.421148680\times10^{+01}$
C_2H_3	2.459276440	$7.371476390\times10^{-03}$	$2.109872870\times10^{-06}$	$-1.321642130\times10^{-09}$	$-1.184783830\times10^{-12}$	$3.335225000\times10^{+04}$	$1.155620190\times10^{+01}$
C_2H_2	2.013562200	$1.519044580\times10^{-02}$	$-1.616318880\times10^{-05}$	$9.078991780\times10^{-09}$	$-1.912746000\times10^{-12}$	$2.612444340\times10^{+04}$	8.805377960
C_2H	3.050667760	$6.051674490\times10^{-03}$	$-4.956634260\times10^{-06}$	$2.804159130\times10^{-09}$	$-8.193332080\times10^{-13}$	$6.630010940\times10^{+04}$	5.954360960
C_3H_6	1.493307110	$2.092517540\times10^{-02}$	$4.486793840\times10^{-06}$	$-1.668912120\times10^{-08}$	$7.158146470\times10^{-12}$	$1.074826420\times10^{+03}$	$1.614534000\times10^{+01}$
C_3H_8	$8.969208000\times10^{-01}$	$2.668986100\times10^{-02}$	$5.431425050\times10^{-06}$	$-2.126000710\times10^{-08}$	$9.243330060\times10^{-12}$	$-1.395491800\times10^{+04}$	$1.935533140\times10^{+01}$

4 Kinetic model parameters calculation

The rates of elementary steps are calculated through the law of mass action with the calculation depending on the nature of the considered process. For reactions involving a gas species i, its density in the gas phase, n_i, is considered, whereas for surface species i, its surface density, ξ_i, is used (see Table S5).

Table S5: Rates calculation of the catalytic model.

Type	Process	Forward rate, r_f $(m^{-2}.s^{-1})$	Backward rate, r_b $(m^{-2}.s^{-1})$	Units of rate constants, k_f/k_b
**Molecular adsorption**	$A + * \rightleftharpoons A*$	$k_f \xi_* n_A$	$k_b \xi_A$	$m^3.s^{-1}/s^{-1}$
**Dissociative adsorption**	$A + 2* \rightleftharpoons B * + C*$	$k_f \xi_*^2 n_A$	$k_b \xi_B \xi_C$	$m^5.s^{-1}/m^2.s^{-1}$
**Langmuir-Hinshelwood reactions**	$A*+B* \rightleftharpoons C*+D*$	$k_f \xi_A \xi_B$	$k_b \xi_C \xi_D$	$m^2.s^{-1}/m^2.s^{-1}$
**Eley-Rideal reactions**	$A + B* \rightleftharpoons C * + D$	$k_f n_A \xi_B$	$k_b \xi_C n_D$	$m^3.s^{-1}/m^3.s^{-1}$

The parameters of the kinetic model are obtained using the thermodynamic constraints defined previously and additional considerations presented below. The rate constants of any forward reaction j is calculated using the Arrhenius equation:

$$k_j^f = k_{0,j}\, exp(-\frac{E_j}{RT_0}) \tag{6}$$

The backward rate constant is obtained via:

$$k_j^b = \frac{k_j^f}{K_{eq,j}} \tag{7}$$

where $K_{eq,j}$ is the equilibrium constant of the reaction for which it holds:

$$K_{eq,j} = exp(\frac{\Delta S_{r,j}}{R} - \frac{\Delta H_{r,j}}{RT_0}) \tag{8}$$

Pre-exponential factors:

Pre-exponential factors $k_{0,j}^{f}$ $(s^{-1}.Pa^{-1})$ of species i colliding with the surface via process j, are calculated using collision theory considering an empty surface[18]. The approach is applied for all adsorption processes and Eley-Rideal reactions:

$$k_{0,j}^{f} = \frac{1}{\xi_{tot}\sqrt{2\pi M_i k_b T_0}} \tag{9}$$

The pre-exponential factor calculated as above corresponds to an upper limit, which is further corrected with a sticking coefficient, s, that represents the probability of the collision being successful[18]. For all adsorption processes a sticking coefficient of 0.1 is used while for the case of Eley-Rideal steps a value of 1 considered. The pre-exponential factors for desorption and reverse Eley-Rideal processes are obtained from equation (1), with the same sticking coefficients applied, so that thermodynamic consistency is upheld.

Pre-exponential factors for forward and backward reactions involving 2 adsorbed species, are estimated using transition state theory and entropic consistency. Specifically, the reaction entropy of each elementary step is evenly distributed around the transition state theory order of magnitude of Langmuir-Hinselwood reactions ($10^{11}\,s^{-1}$) to satisfy equation (1).

Activation energies and reaction enthalpies:

Dissociative adsorptions are activated as they involve the cleavage of a covalent bond. For species A that adsorbs via $A + 2* \rightleftharpoons B* + C*$ the forward activation energy is obtained from the unity bond index-quadratic exponential potential (UBI-QEP) method[24,25]:

$$E_{ads_j}^{f} = \frac{1}{2}\left[\Delta H_{ads,j} - \chi_A + \frac{\chi_B \chi_C}{\chi_B + \chi_C}\right] \tag{10}$$

where $\Delta H_{ads,j}$ is the enthalpy of adsorption estimated from:

$$\Delta H_{ads,j} = H_{s,B} + H_{s,C} - H_{g,A} \tag{11}$$

Molecular adsorptions ($A + * \rightleftharpoons A*$) do not lead to bond cleavage and consequently are assumed to be non-activated. Consequently, the forward activation energy, ${E_{ads}}_j^f$, is set to 0.

For the case of surface reactions ($A * + B* \rightleftharpoons C * + D*$), the activation energy of the forward step is again calculated using the UBI-QEP method according to:

$$ {E_{LH}}_j^f = \frac{1}{2}\left[\mathit{\Delta} H_{s,j} + \frac{\chi_C \chi_D}{\chi_C + \chi_D}\right] \tag{12} $$

where the surface reaction enthalpy is calculated based on the heats of chemisorption of the participating species and the reaction enthalpy of the equivalent gas phase reaction ($A + B \rightleftharpoons C + D$):

$$ \mathit{\Delta} H_{s,j} = \Delta H_{g,j} + \chi_A + \chi_B - \chi_C - \chi_D \tag{13} $$

The activation energies of hydrogen transfer steps, namely the Eley-Rideal processes considered in this work ($A * + BH \rightleftharpoons B + AH*$), are estimated using the Polanyi-Semenov equation that was proposed by Krylov in [26] by first determining the activation energy of the forward process, ${E_{ER}}_j^f$:

$$ {E_{ER}}_j^f = E_0 + 0.75\,\Delta H_{ER,j} \tag{14} $$

where $E_0 = 30\,kJ.mol^{-1}$ and the enthalpy of the step $\Delta H_{ER,j}$ is:

$$ \Delta H_{ER,j} = H_{g,B} + H_{s,AH} - H_{s,A} - H_{g,BH} \tag{15} $$

The activation energy of the reverse process, ${E_{ER}}_j^b$ is obtained from Eq 2. If ${E_{ER}}_j^b$ is found negative, the reverse reaction is assumed to be non-activated and its activation energy is set to zero, while ${E_{ER}}_j^f$ is set equal to $\Delta H_{ER,j}$ to satisfy Eq 2. Most of these processes are found to be non-activated in the direction of the abstraction of an H from the surface species by the gas one (see part 7 below).

Reactivity of vibrationally excited states:

The energy contained in vibrationally excited states of methane, ethane and hydrogen is assumed to enhance the rate of the non-reversible adsorption of these species on catalyst sites, by decreasing the activation barrier of the equivalent ground state process proportionally to the excitation energy, E_ν. This mechanism is discussed in several literature works[7,27], and is considered to be contributing to positive performances obtained with plasma-catalysis of methane upgrading[3,4], and other processes, such as ammonia production[28–30].

The approach employed here follows the Fridman-Macheret model[31] that was employed for the reactivity of vibrationally excited species in the gas phase[16]. The coefficient α is calculated using the forward and backward activation energies of the ground-state adsorption at a given temperature:

$$\alpha = \frac{E^f}{E^f + E^b} \tag{16}$$

This coefficient α is then used as a scaling factor to reduce the forward activation barrier of the excited state dissociative adsorption, $E^f(vib)$, such that:

$$E^f(vib) = E^f - \alpha\, E_\nu \tag{17}$$

The reduction of the forward activation barrier leads to a significant increase of the forward rate constant of adsorption (the values for ethane and hydrogen are available in the next section). Indeed, $CH_4(\nu 2,4)$ and $CH_4(\nu 1,3)$ exhibit rates constants of adsorption 5.64 and 47.7 times higher than CH_4, respectively (Table S6). Nonetheless, as seen in[16], VT and VV processes are enhanced by products and higher temperatures, hence the population of $CH_4(\nu 1,3)$ remains much smaller than $CH_4(\nu 2,4)$, which could lead to an overall higher contribution of $CH_4(\nu 2,4)$.

Table S6: Effect of vibrational energy on the adsorption processes of methane, values at 500 K. No backward activation energy is considered for the cases involving $CH_4(\nu 2,4)$ and $CH_4(\nu 1,3)$ as only the ground-state desorbs.

**Process**	E^f **(kJ.mol^{-1})**	E^b **(kJ.mol^{-1})**	$\alpha(-)$	$k_j^f/k_{CH_4}^f(-)$
$CH_4 + 2* \leftrightarrow CH_3* + H*$	41.10	49.31		1.00
$CH_4(\nu 2,4) + 2* \rightarrow CH_3* + H*$	33.91	–	0.45	5.64
$CH_4(\nu 1,3) + 2* \rightarrow CH_3* + H*$	25.03	–		47.7

5 Adsorption processes

The complete set of adsorption processes are detailed here with values at 500 K presented.

5.1 Dissociative processes

Table S7: Dissociative adsorptions considered in the model along with the equivalent processes involving vibrationally excited states.

Process	α(-)	$k^f_{0,j}$ $(Pa^{-1}.s^{-1})$	E^f_j $(kJ.mol^{-1})$	E^b_j $(kJ.mol^{-1})$	$\Delta H_{s,j}$ $(kJ.mol^{-1})$	$\Delta S_{s,j}$ $(J.mol^{-1}.K^{-1})$
$CH_4 + 2* \rightleftharpoons CH_3 * + H*$	0.4546	2.942×10^3	41.10	49.31	-8.215	-139.13
$CH_4(\nu2,4) + 2* \rightarrow CH_3 * + H*$		2.942×10^3	33.91	-	-	-
$CH_4(\nu1,3) + 2* \rightarrow CH_3 * + H*$		2.942×10^3	25.03	-	-	-
$C_2H_6 + 2* \rightleftharpoons C_2H_5 * + H*$	0.5839	2.149×10^3	37.10	26.44	10.66	-137.61
$C_2H_6(\nu2,4) + 2* \rightarrow C_2H_5 * + H*$		2.149×10^3	27.98	-	-	-
$C_2H_6(\nu1,3) + 2* \rightarrow C_2H_5 * + H*$		2.149×10^3	15.95	-	-	-
$H_2 + 2* \rightleftharpoons H * + H*$	0.087	8.291×10^3	8.037	84.12	-76.09	-134.38
$H_2\nu(1) + 2* \rightarrow H * + H*$		8.291×10^3	3.780	-	-	-
$H_2\nu(2) + 2* \rightarrow H * + H*$		8.291×10^3	0	-	-	-
$H_2\nu(3) + 2* \rightarrow H * + H*$		8.291×10^3	0	-	-	-

5.2 Molecular adsorptions

Table S8: Molecular adsorptions considered in the model.

Process	$k^f_{0,j}(Pa^{-1}.s^{-1})$	E^f_j $(kJ.mol^{-1})$	E^b_j $(kJ.mol^{-1})$	$\Delta H_{s,j}$ $(kJ.mol^{-1})$	$\Delta S_{s,j}$ $(J.mol^{-1}.K^{-1})$
$CH_3 + * \rightleftharpoons CH_3*$	3.039×10^3	0	193.92	-193.92	-153.40
$CH_2 + * \rightleftharpoons CH_2*$	3.147×10^3	0	426.24	-426.24	-152.53
$CH + * \rightleftharpoons CH*$	3.267×10^3	0	639.36	-639.36	-151.60
$C + * \rightleftharpoons C*$	3.401×10^3	0	674.88	-674.88	-150.59
$C_2H_5 + * \rightleftharpoons C_2H_5*$	2.186×10^3	0	153.60	-153.60	-161.62
$C_2H_4 + * \rightleftharpoons C_2H_4*$	2.225×10^3	0	82.56	-82.56	-161.18
$C_2H_3 + * \rightleftharpoons C_2H_3*$	2.266×10^3	0	294.72	-294.72	-160.72
$C_2H_2 + * \rightleftharpoons C_2H_2*$	2.310×10^3	0	251.52	-251.52	-160.25
$C_2H + * \rightleftharpoons C_2H*$	2.356×10^3	0	512.64	-512.64	-159.75
$H + * \rightleftharpoons H*$	1.514×10^4	0	257.28	-257.28	-119.73

6 Surface reactions

The surface reactions following the Langmuir-Hinshelwood formalism are detailed here with data presented at 500 K.

Table S9: Surface reactions reactions following the Langmuir-Hinshelwood formalism considered in the model.

Process	$k_{0,j}^{f}$ (s^{-1})	E_j^f $(kJ.mol^{-1})$	E_j^b $(kJ.mol^{-1})$	$\Delta H_{s,j}$ $(kJ.mol^{-1})$	$\Delta S_{s,j}$ $(J.mol^{-1}.K^{-1})$
$CH_3*+* \rightleftharpoons CH_2*+H*$	$8.749\times10^{+03}$	63.58	96.86	-33.28	-140.89
$CH_2*+* \rightleftharpoons CH*+H*$	$1.616\times10^{+03}$	67.62	115.84	-48.22	-154.93
$CH_2*+CH* \rightleftharpoons CH_3*+C*$	$7.212\times10^{+09}$	114.10	36.54	77.56	-27.32
$CH_2*+C* \rightleftharpoons CH*+CH*$	$1.663\times10^{+11}$	113.59	206.09	-92.50	13.28
$CH*+* \rightleftharpoons C*+H*$	$3.273\times10^{+02}$	115.27	71.00	44.28	-168.21
$CH_3*+CH_2* \rightleftharpoons C_2H_4*+H*$	$3.698\times10^{+09}$	39.88	22.62	17.27	-33.02
$C_2H_4*+* \rightleftharpoons CH_2*+CH_2*$	$4.644\times10^{+05}$	81.29	131.84	-50.55	-107.87
$C_2H_5*+CH_2* \rightleftharpoons C_2H_4*+CH_3*$	$3.671\times10^{+09}$	27.87	30.04	-2.17	-33.09
$C_2H_3*+CH_2* \rightleftharpoons C_2H_2*+CH_3*$	$7.044\times10^{+09}$	43.08	66.42	-23.34	-27.52
$C_2H*+CH_2* \rightleftharpoons C_2H_2*+CH*$	$2.310\times10^{+10}$	51.21	129.30	-78.10	-16.93
$C_2H_5*+C_2H* \rightleftharpoons C_2H_4*+C_2H_2*$	$2.609\times10^{+09}$	0.000	65.33	-65.33	-35.97
$C_2H_3*+C_2H_3* \rightleftharpoons C_2H_4*+C_2H_2*$	$2.038\times10^{+09}$	13.089	49.068	-35.98	-38.046
$C_2H_3*+C_2H_2* \rightleftharpoons C_2H_4*+C_2H*$	$5.703\times10^{+10}$	60.812	10.296	50.52	-7.641
$C_2H_3*+C_2H* \rightleftharpoons C_2H_2*+C_2H_2*$	$5.032\times10^{+09}$	19.632	106.128	-86.50	-30.406
$C_2H_5*+* \rightleftharpoons C_2H_4*+H*$	$1.636\times10^{+02}$	13.522	48.981	-35.46	-173.975
$C_2H_4*+* \rightleftharpoons C_2H_3*+H*$	$3.101\times10^{+04}$	58.362	79.003	-20.64	-130.366
$C_2H_3*+* \rightleftharpoons C_2H_2*+H*$	$3.194\times10^{+02}$	35.282	91.902	-56.62	-168.412
$C_2H_2*+* \rightleftharpoons C_2H*+H*$	$1.237\times10^{+04}$	100.591	70.715	29.88	-138.006

7 Eley-Rideal processes

The Eley-Rideal processes considered in the model are detailed in Table S10 with data presented at 500 K. The reactions are written in the dominant direction of hydrogen abstraction from an adsorbed species. For all the reverse processes, where a molecule (CH_4 ,C_2H_6, C_2H_4, C_2H_2, C_3H_8, C_3H_6) looses an hydrogen onto an adsorbed species, the equivalent processes involving vibrationally excited modes of the molecules were accounted for, under the assumption that the same rate as the ground-state applies.

Table S10: Surface reactions following an Eley-Rideal formalism considered.

Process	$k_{0,j}^f$ ($Pa^{-1}.s^{-1}$)	E_j^f ($kJ.mol^{-1}$)	E_j^b ($kJ.mol^{-1}$)	$\Delta H_{s,j}$ ($kJ.mol^{-1}$)	$\Delta S_{s,j}$ ($J.mol^{-1}.K^{-1}$)
$CH_3 + CH_3* \rightleftharpoons CH_2* + CH_4$	3.039×10^3	0.0	213.11	-213.11	-9.53
$CH_3 + CH_2* \rightleftharpoons CH* + CH_4$	3.039×10^3	0.0	228.05	-228.05	-23.57
$CH_3 + CH* \rightleftharpoons C* + CH_4$	3.039×10^3	0.0	135.55	-135.55	-36.85
$CH_2 + CH_3* \rightleftharpoons CH_2* + CH_3$	3.147×10^3	0.0	232.32	-232.32	0.868
$CH_2 + CH_2* \rightleftharpoons CH* + CH_3$	3.147×10^3	0.0	247.26	-247.26	-13.17
$CH_2 + CH* \rightleftharpoons C* + CH_3$	3.147×10^3	0.0	154.76	-154.76	-26.45
$CH + CH_3* \rightleftharpoons CH_2* + CH_2$	3.267×10^3	0.0	198.18	-198.18	14.98
$CH + CH_2* \rightleftharpoons CH* + CH_2$	3.267×10^3	0.0	213.12	-213.12	0.932
$CH + CH* \rightleftharpoons C* + CH_2$	3.267×10^3	0.0	120.62	-120.62	-12.35
$C + CH_3* \rightleftharpoons CH_2* + CH$	3.401×10^3	1.729	114.81	-113.08	28.33
$C + CH_2* \rightleftharpoons CH* + CH$	3.401×10^3	0.0	128.02	-128.02	14.29
$C + CH* \rightleftharpoons C* + CH$	3.401×10^3	21.12	56.640	-35.52	1.008
$C_2H_5 + CH_3* \rightleftharpoons CH_2* + C_2H_6$	2.186×10^3	0.0	191.67	-191.67	-19.26
$C_2H_5 + CH_2* \rightleftharpoons CH* + C_2H_6$	2.186×10^3	0.0	206.60	-206.60	-33.30
$C_2H_5 + CH* \rightleftharpoons C* + C_2H_6$	2.186×10^3	1.474	115.58	-114.10	-46.58
$C_2H_3 + CH_3* \rightleftharpoons CH_2* + C_2H_4$	2.267×10^3	0.0	224.80	-224.80	-10.07
$C_2H_3 + CH_2* \rightleftharpoons CH* + C_2H_4$	2.267×10^3	0.0	239.74	-239.74	-24.11
$C_2H_3 + CH* \rightleftharpoons C* + C_2H_4$	2.267×10^3	0.0	147.24	-147.24	-37.39
$C_2H + CH_3* \rightleftharpoons CH_2* + C_2H_2$	2.356×10^3	0.0	324.28	-324.28	-2.39
$C_2H + CH_2* \rightleftharpoons CH* + C_2H_2$	2.356×10^3	0.0	339.22	-339.22	-16.43
$C_2H + CH* \rightleftharpoons C* + C_2H_2$	2.356×10^3	0.0	246.72	-246.72	-29.71
$C_3H_5 + CH_3* \rightleftharpoons CH_2* + C_3H_6$	1.840×10^3	0.0	121.75	-121.75	49.32
$C_3H_5 + CH_2* \rightleftharpoons CH* + C_3H_6$	1.840×10^3	0.0	136.69	-136.69	35.28
$C_3H_5 + CH* \rightleftharpoons C* + C_3H_6$	1.840×10^3	18.95	63.14	-44.19	22.00
$C_3H_7 + CH_3* \rightleftharpoons CH_2* + C_3H_8$	1.797×10^3	0.0	163.86	-163.86	33.33
$C_3H_7 + CH_2* \rightleftharpoons CH* + C_3H_8$	1.797×10^3	0.0	178.80	-178.80	19.29
$C_3H_7 + CH* \rightleftharpoons C* + C_3H_8$	1.797×10^3	8.46	94.73	-86.30	6.01

8 Density profiles of CH_4 and C_2 species in linear scale

Number densities of CH_4 and its excited states and of C_2 products, as presented in Figure 2 of the main manuscript, are provided in Figure S1 in linear scale for the different cases simulated to demonstrate more clearly the approach to steady state.

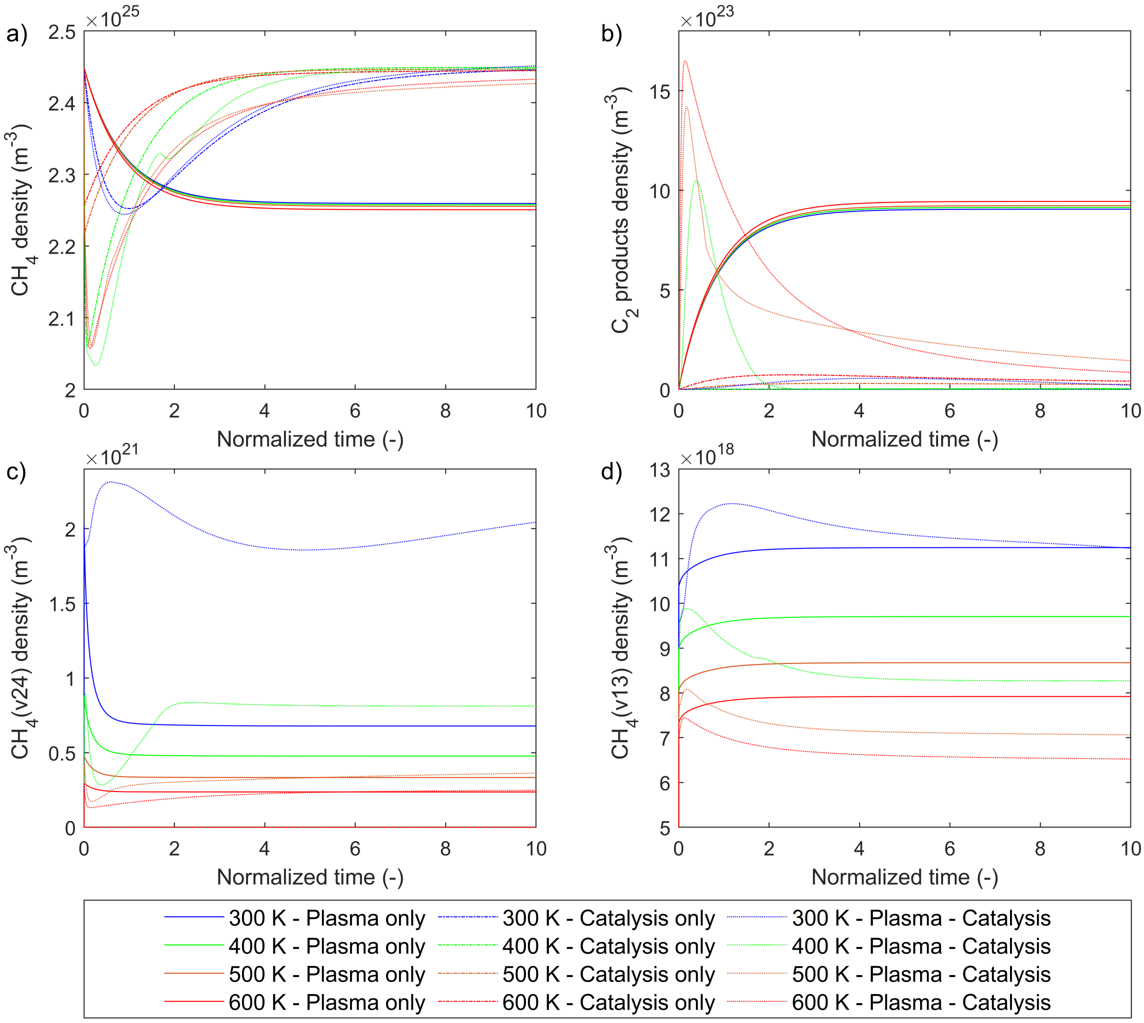

Figure S1: Gas species number densities (m^{-3}) against normalised time in linear scale for the different cases simulated. a) CH_4, b) C_2 species, c) $CH_4(\nu 2, 4)$, d) $CH_4(\nu 1, 3)$.

9 Density profiles of main radicals

The densities of the radicals with the highest populations for all cases and temperatures studied are presented in Figure S2. For all radicals, the densities are highest for plasma-only cases, followed by plasma-catalysis, while for catalysis-only values are significantly lower. The profiles of H and CH_3 radicals display much less variation with time in comparison to those of methane and C_2 species (Figure 2 in main manuscript) on account of their high reactivity and their rapid

formation and consumption. The density of C_2H_5 follows the formation of ethane, with the profile resembling that of C_2 presented in the main manuscript. At 600 and 500 K the density of radicals in the catalysis-only case is seen to increase due to the thermal activation of methane.

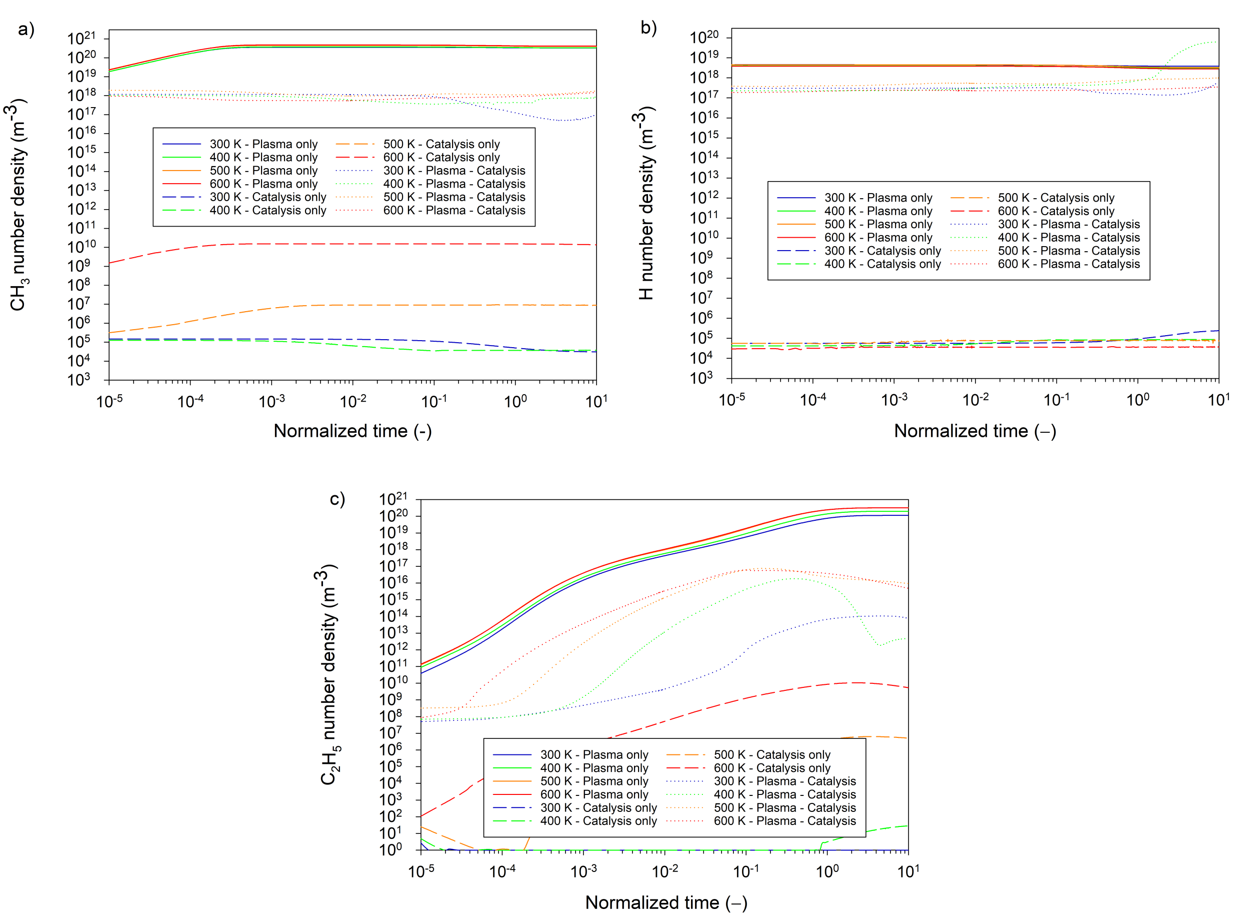

Figure S2: Densities of the most populated radicals for all cases and temperatures studied, a) CH_3, b) H, c) C_2H_5

10 Product selectivities and methane conversion

The selectivities of all products obtained at maximum methane conversion of each temperature are given in Table S11. As the catalyst retains in cases up to 10% of total carbon (e.g. at 300 K), values reported are based on product densities according to:

$$S_{C_xH_y} = \frac{n_{C_xH_y}}{\sum n_{products}}$$

For each reactor case, the selectivities towards the C_2 and C_3 species showed minimal variation for the studied temperatures. Catalysis cases were selective only towards ethane, whereas

traces of ethylene were observed for plasma-catalysis cases, originating mainly from the dehydrogenation of ethane by electron collision processes. Only a small effect of temperature was observed for the plasma-only case, with conversion varying minimally, and selectivities being almost identical, between 300 to 600 K. Both catalysis cases show similar results of maximum methane conversion at 300 K, with larger deviations observed at higher temperatures. The highest conversion of the catalysis-only and plasma-catalysis cases at 400 K corresponds to the situation where methane adsorption/desorption is unbalanced and the catalyst mainly acts as a sink of methane, without emitting significant amounts of products. At 500 K the conversion in the catalysis-only scenario is higher than at 600 K as at the higher temperature adsorption/desorption cycles are faster, resulting at lower values that the densities of CH_3* and $H*$ stabilise at. The plasma-catalysis conversion at 500 and 600 K are similar and about 2 times higher than the equivalent plasma-only cases. These are the optimal results that are attained in these simulations, however, as discussed in the main manuscript, they are only momentary values that diminish following the adsorption of H_2 onto the catalyst.

Table S11: Selectivities of C_2 and C_3 at maximum methane conversion, for all scenarios and temperatures considered.

	Temperature (K)	Max X_{CH_4} (%)	$S_{C_2H_6}$(%)	$S_{C_2H_4}$(%)	$S_{C_2H_2}$(%)	$S_{C_3H_8}$(%)	$S_{C_3H_6}$(%)
Plasma-only	300	7.780	27.454	51.483	19.541	0.480	1.039
	400	7.859	27.375	52.256	19.075	0.440	0.851
	500	7.945	27.238	52.565	19.003	0.411	0.780
	600	8.138	27.000	52.801	19.058	0.393	0.745
Catalysis-only	300	8.071	~100	9.360×10^{-11}	8.753×10^{-11}	8.430×10^{-10}	8.779×10^{-10}
	400	15.903	~100	9.386×10^{-12}	3.469×10^{-15}	6.482×10^{-14}	7.170×10^{-14}
	500	9.503	~100	6.016×10^{-10}	4.929×10^{-16}	7.937×10^{-15}	5.470×10^{-15}
	600	7.966	~100	4.466×10^{-09}	9.880×10^{-17}	7.179×10^{-15}	3.0211×10^{-15}
Plasma-catalysis	300	8.402	99.999	3.9837×10^{-04}	2.0453×10^{-09}	4.3717×10^{-05}	1.3266×10^{-04}
	400	16.36	99.993	4.4721×10^{-04}	3.3847×10^{-08}	1.3106×10^{-03}	2.7483×10^{-03}
	500	15.92	99.997	5.3138×10^{-04}	1.5604×10^{-08}	8.0699×10^{-04}	1.1397×10^{-03}
	600	16.03	99.997	2.6007×10^{-04}	6.4998×10^{-09}	7.1140×10^{-04}	1.0270×10^{-03}

11 Temperature effect on surface densities

The surface species densities in catalysis-only and plasma-catalysis scenarios at 300, 400 and 600 K are presented in Figures S3, S4 and S5, respectively. Qualitatively identical trends to those at 500 K presented in the main manuscript are visible for both cases. The initial stabilisation of CH_3* and $H*$ occurs faster at higher temperatures, while the respective densities are higher at lower temperatures, both due to the faster adsorption/desorption of methane at higher temperature. A slight increase of $H*$ at 600 K for the catalysis-only case is a consequence of methane thermal activation: $H + CH_4 \rightarrow CH_3 + H_2$ accelerating and creating molecular hydrogen that dissociatively adsorbs on the catalyst. The plasma-catalysis cases all are characterised by the same divergence of CH_3* and $H*$, after the initial stabilisation and the onset of ethane production. For the plasma-catalysis case, the highest coverage is observed at 400 K due to the unbalanced adsorption/desorption cycles discussed in the main manuscript.

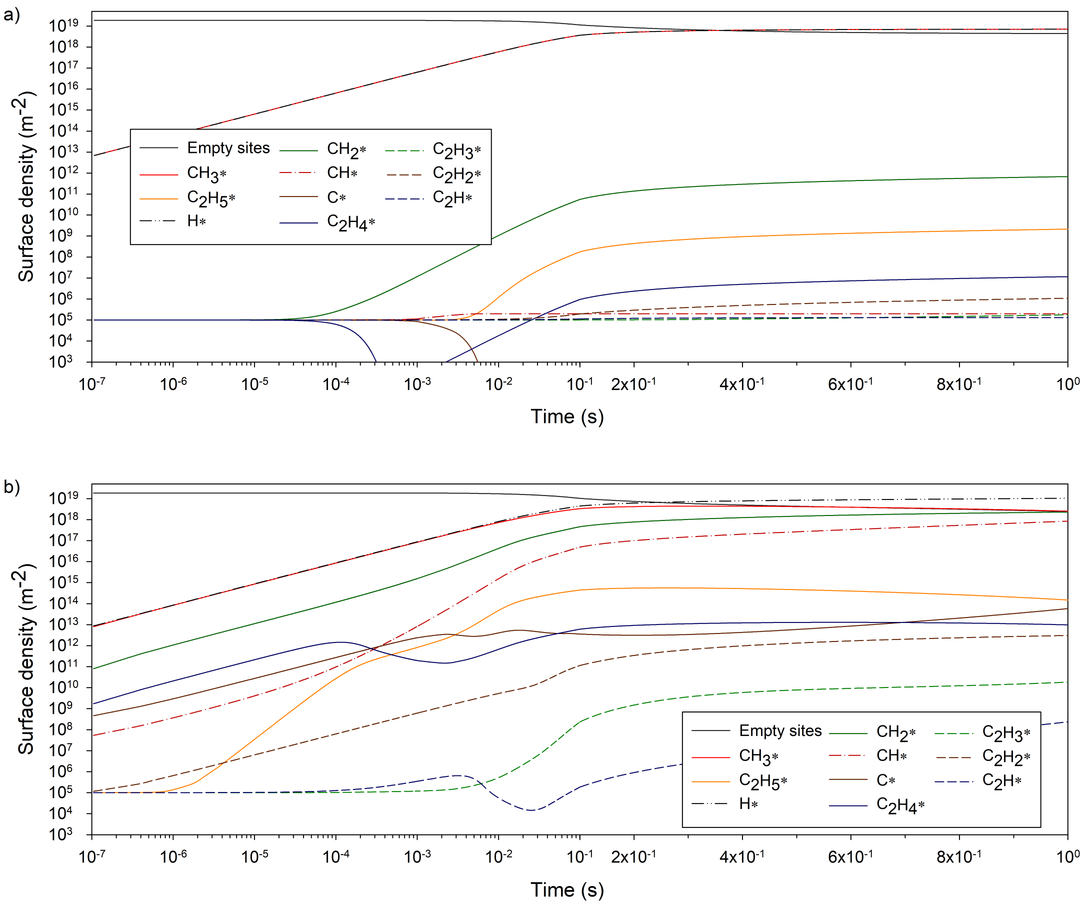

Figure S3: Surface densities (m^{-2}) over time (s) at 300 K. a) Catalysis-only, b) Plasma-catalysis

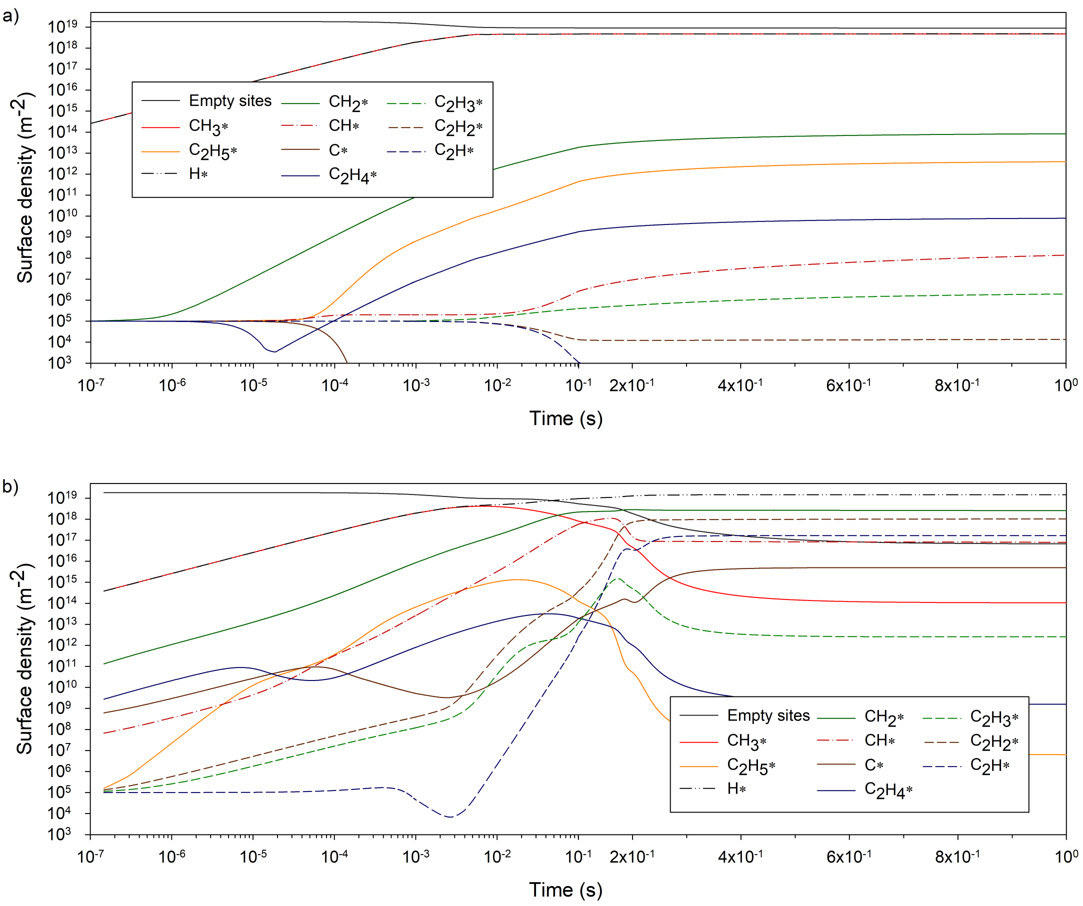

Figure S4: Surface densities (m^{-2}) over time (s) at 400 K. a) Catalysis-only, b) Plasma-catalysis

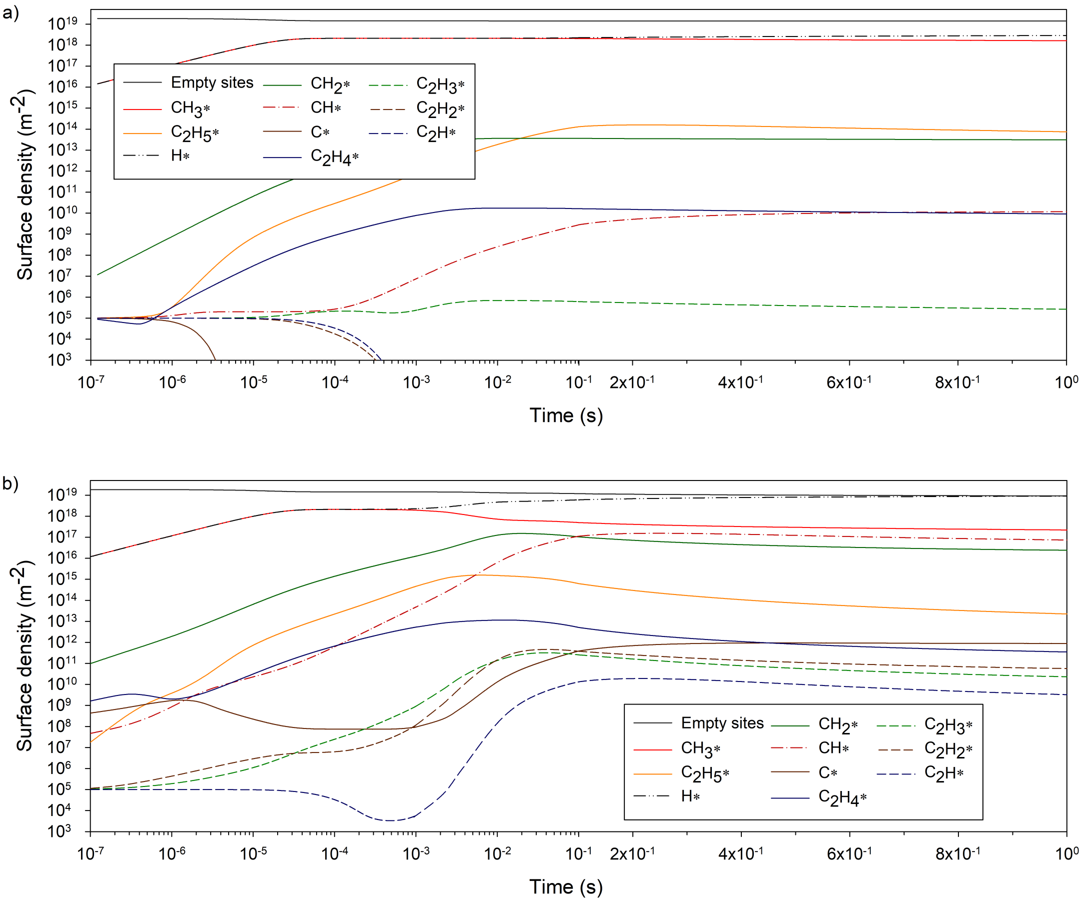

Figure S5: Surface densities (m^{-2}) over time (s) at 600 K. a) Catalysis-only, b) Plasma-catalysis

12 Effect of temperature on the reaction pathways of C_2H_4* and C_2H_5*

The reaction pathway analysis results on C_2H_4* and $C_2H_5^*$, despite the important role of the species in plasma-catalysis, showed little variation with temperature (Figures S6 and S7). At all temperatures, the population of C_2H_4* is rather stable, with the species having a very low net rate of production, several orders of magnitude lower than its production and consumption. Its production is largely unaffected by temperature and is dominated ethylene adsorption ($C_2H_4 + * \rightarrow C_2H_4*$) and surface coupling $CH_3 * + CH_2* \rightarrow C_2H_4 * + H*$). C_2H_4* mainly hydrogenates towards C_2H_5* via surface H-transfer ($C_2H_4 * + CH_3* \rightarrow C_2H_5 * + CH_2*$). At higher temperature, due to the adsorption of H_2, the direct hydrogenation of C_2H_4* into C_2H_5* with $H*$ becomes significant. At maximum methane conversion, the net rate of C_2H_5* production

overlaps with its rate of production, the latter being driven by the same processes responsible for C_2H_4* consumption. Only a negligible impact of the excited states of ethane $C_2H_6(\nu1,3)$ and $C_2H_6(\nu2,4)$ is visible at high temperature. At all temperatures, the net consumption of C_2H_5* is dominated by its desorption as ethane.

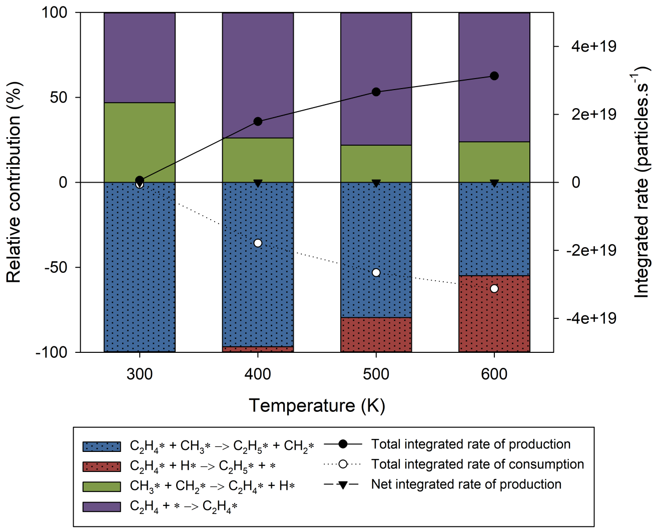

Figure S6: Reaction pathway analysis of C_2H_4*. Relative contribution (%) and total integrated rates ($particles.s^{-1}$) over temperature (K).

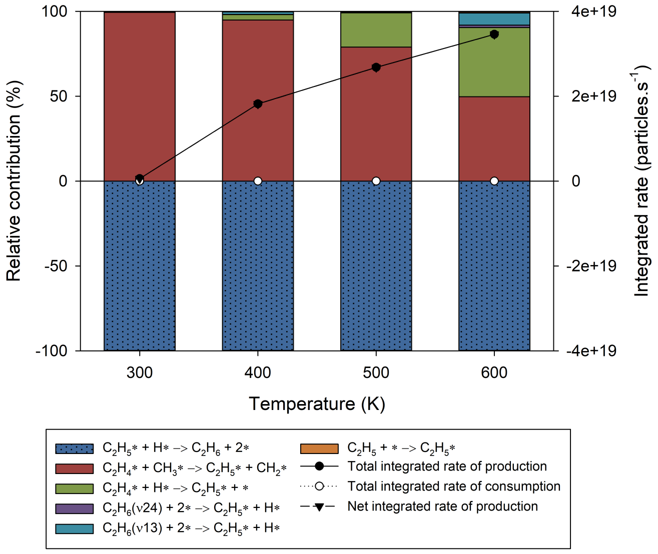

Figure S7: Reaction pathway analysis of C_2H_5*. Relative contribution (%) and total integrated rates ($particles.s^{-1}$) over temperature (K).

13 Hydrogen transformations in plasma-catalysis

Hydrogen transformations for plasma-catalysis at 500 K at peak simulation time $t_{start} = 0.018\, s$ are presented in Figure S8. The dissociation of methane via electron collisions is the main source of H radicals in the gas phase. The latter are very short-lived species and mostly adsorb on the catalyst (90.89% of its total consumption). $H*$ is formed via the dissociative adsorption of methane and molecular hydrogen H_2. The latter is even faster than the excitation of H_2 to $H_2\nu(1)$, $H_2\nu(2)$ and $H_2\nu(3)$, as visible from their minute contribution to the consumption of H_2. Ethane is the main source of H_2 through electron collisions, confirming the propensity of the catalyst to loose activity as ethane is formed (discussed in the main manuscript).

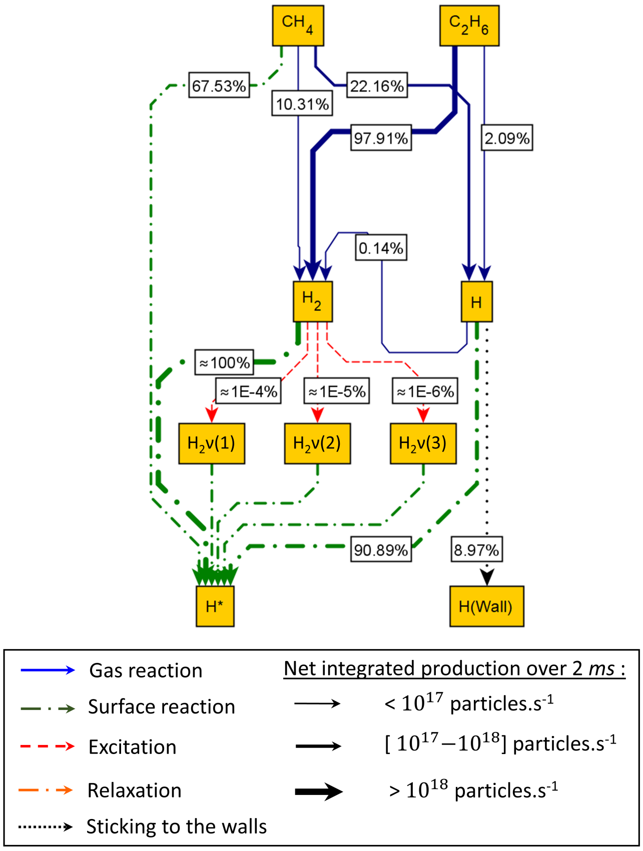

Figure S8: Reaction pathway analysis of hydrogen at 500 K and at maximum CH_4 consumption for plasma-catalysis.

14 Reaction enthalpies considered in the energy efficiency calculations

The thermodynamic data used used for the energy efficiency calculations are collected in Tables S12 and S13. The enthaply of formation of all molecules at gas temperature T_0, $H_f^{T_0}$, is obtained from the NASA chemical equilibrium polynomials[23] provided in Section 3, based on which the enthalpies of reaction $\Delta H_r^{T_0}$ are also obtained.

Table S12: Enthalpy of formation ($H_f^{T_0}$, $kJ.mol^{-1}$) at the temperatures studied.

Species	Temperature (K)			
	300	400	500	600
CH_4	-74.83	-70.10	-66.61	-61.68
C_2H_6	-83.76	-77.84	-70.66	-62.30
C_2H_4	52.54	57.38	63.19	69.84
C_2H_2	226.85	231.59	236.85	242.51
C_3H_8	-103.72	-95.30	-84.96	-72.89
C_3H_6	20.57	27.85	36.65	46.82
H_2	0.056	2.96	5.88	8.81
C	716.71	718.80	720.88	722.96

Table S13: Enthalpy of reaction ($\Delta H_r^{T_0}$, $kJ.mol^{-1}$) at the temperatures studied.

Processes	Temperature (K)			
	300	400	500	600
$CH_4 \rightarrow 0.5C_2H_6 + 0.5H_2$	32.98	33.56	34.22	34.94
$CH_4 \rightarrow 0.5C_2H_4 + H_2$	101.16	102.64	104.08	105.41
$CH_4 \rightarrow 0.5C_2H_2 + 1.5H_2$	188.34	191.23	193.85	196.15
$CH_4 \rightarrow \frac{1}{3}C_3H_8 + \frac{2}{3}H_2$	40.29	41.20	42.21	43.26
$CH_4 \rightarrow \frac{1}{3}C_3H_6 + H_2$	81.74	83.24	84.70	86.10
$CH_4 \rightarrow C + 2H_2$	791.66	795.71	799.24	802.26

References

(1) Ross, J. R. H. *Heterogeneous Catalysis*; Elsevier, 2012.

(2) Nozaki, T.; Okazaki, K. Non-thermal plasma catalysis of methane: Principles, energy efficiency, and applications. *Catalysis Today* **2013**, *211*, 29–38.

(3) Loenders, B.; Engelmann, Y.; Bogaerts, A. Plasma-Catalytic Partial Oxidation of Methane on Pt(111): A Microkinetic Study on the Role of Different Plasma Species. *Journal of Physical Chemistry C* **2021**,

(4) Engelmann, Y.; Mehta, P.; Neyts, E. C.; Schneider, W. F.; Bogaerts, A. Predicted Influence of Plasma Activation on Nonoxidative Coupling of Methane on Transition Metal Catalysts. *ACS Sustainable Chemistry & Engineering* **2020**, *8*, 6043–6054.

(5) Sheng, Z.; Watanabe, Y.; Kim, H.-H.; Yao, S.; Nozaki, T. Plasma-enabled mode-selective activation of CH_4 for dry reforming: First touch on the kinetic analysis. *Chemical Engineering Journal* **2020**, *399*.

(6) Whitehead, J. C. Plasma-catalysis: Is it just a question of scale? *Frontiers of Chemical Science and Engineering* **2019**, *13*, 264–273.

(7) Bogaerts, A.; Zhang, Q.-Z.; Zhang, Y.-R.; Van Laer, K.; Wang, W. Burning questions of plasma catalysis: Answers by modeling. *Catalysis Today* **2019**, *337*, 3–14.

(8) Mehta, P.; Barboun, P.; Go, D.; Hicks, J.; Schneider, W. Catalysis Enabled by Plasma Activation of Strong Chemical Bonds: A Review. *ACS Energy Letters* **2019**, *4*, 1115–1133.

(9) Neyts, E. C.; Bogaerts, A. Understanding plasma catalysis through modelling and simulation—a review. *Journal of Physics D: Applied Physics* **2014**, *47*, 224010.

(10) Zhang, Q.-Z.; Bogaerts, A. Plasma streamer propagation in structured catalysts. *Plasma Sources Science and Technology* **2018**,

(11) Zhang, Q.-Z.; Bogaerts, A. Propagation of a plasma streamer in catalyst pores. *Plasma Sources Science and Technology* **2018**, *27*, 035009.

(12) Snoeckx, R.; Aerts, R.; Tu, X.; Bogaerts, A. Plasma-Based Dry Reforming: A Computational Study Ranging from the Nanoseconds to Seconds Time Scale. *The Journal of Physical Chemistry C* **2013**, *117*, 4957–4970.

(13) Heijkers, S.; Aghaei, M.; Bogaerts, A. Plasma-Based CH_4 Conversion into Higher Hydrocarbons and H_2 : Modeling to Reveal the Reaction Mechanisms of Different Plasma Sources. *Journal of Physical Chemistry C* **2020**, *124*, 7016–7030.

(14) Chen, X.; Zhang, S.; Li, S.; Zhang, C.; Pan, J.; Murphy, A. B.; Shao, T. Temperature-independent, nonoxidative methane conversion in nanosecond repetitively pulsed DBD plasma. *Sustainable Energy Fuels* **2021**, *5*, 787–800.

(15) De Bie, C.; Verheyde, B.; Martens, T.; van Dijk, J.; Paulussen, S.; Bogaerts, A. Fluid Modeling of the Conversion of Methane into Higher Hydrocarbons in an Atmospheric Pressure Dielectric Barrier Discharge. *Plasma Processes and Polymers* **2011**, *8*, 1033–1058.

(16) Maitre, P. A.; Bieniek, M. S.; Kechagiopoulos, P. N. Modelling excited species and their role on kinetic pathways in the non-oxidative coupling of methane by dielectric barrier discharge. *Chemical Engineering Science* **2021**, *234*, 116399.

(17) Mhadeshwar, A. B.; Wang, H.; Vlachos, D. G. Thermodynamic Consistency in Microkinetic Development of Surface Reaction Mechanisms. *Journal of Physical Chemistry B* **2003**, *107*, 12721–12733.

(18) Dumesic, J. A. *The Microkinetics of heterogeneous catalysis*; ACS professional reference book; American Chemical Society: Washington, DC, 1993.

(19) Kechagiopoulos, P.; Angeli, S.; Lemonidou, A. Low temperature steam reforming of methane: A combined isotopic and microkinetic study. *Applied Catalysis B: Environmental* **2017**, *205*, 238–253.

(20) Santiago, M.; Sánchez-Castillo, M.; Cortright, R.; Dumesic, J. Catalytic Reduction of Acetic Acid, Methyl Acetate, and Ethyl Acetate over Silica-Supported Copper. *Journal of Catalysis* **2000**, *193*, 16–28.

(21) Kandoi, S.; Greeley, J.; Simonetti, D.; Shabaker, J.; Dumesic, J. A.; Mavrikakis, M. Reaction Kinetics of Ethylene Glycol Reforming over Platinum in the Vapor versus Aqueous Phases. *Journal of Physical Chemistry C* **2011**, *115*, 961–971.

(22) Kandoi, S.; Greeley, J.; Sanchez-Castillo, M. A.; Evans, S. T.; Gokhale, A. A.; Dumesic, J. A.; Mavrikakis, M. Prediction of Experimental methanol Decomposition Rates on Platinum from First Principles. *Topics in Catalysis* **2006**, *37*, 17–28.

(23) Gordon, S.; McBride, B. J.; Lewis Research Center.,; United States., *Computer program for calculation of complex chemical equilibrium compositions, rocket performance, incident and reflected shocks, and Chapman-Jouguet detonations*; NASA SP.273; Scientific and Technical Information Office, National Aeronautics and Space Administration: Washington, D.C., 1976.

(24) Shustorovich, E. *Advances in Catalysis*; Elsevier, 1990; Vol. 37; pp 101–163.

(25) Shustorovich, E. The UBI-QEP method: A practical theoretical approach to understanding chemistry on transition metal surfaces. *Surface Science Reports* **1998**, *31*, 1–119.

(26) Krylov, O. V. Catalytic reactions of partial methane oxidation. *Catalysis Today* **1993**, *18*, 209–302.

(27) Neyts, E. C.; Ostrikov, K. K.; Sunkara, M. K.; Bogaerts, A. Plasma Catalysis: Synergistic Effects at the Nanoscale. *Chemical Reviews* **2015**, *115*, 13408–13446.

(28) Veer, K. v. t.; Alphen, S. V.; Remy, A.; Gorbanev, Y.; Geyter, N. D.; Snyders, R.; Reniers, F.; Bogaerts, A. Spatially and temporally non-uniform plasmas: microdischarges from the perspective of molecules in a packed bed plasma reactor. *Journal of Physics D: Applied Physics* **2021**,

(29) Veer, K. v. t.; Reniers, F.; Bogaerts, A. Zero-dimensional modeling of unpacked and packed bed dielectric barrier discharges: the role of vibrational kinetics in ammonia synthesis. *Plasma Sources Science and Technology* **2020**, *29*, 045020.

(30) Hong, J.; Pancheshnyi, S.; Tam, E.; Lowke, J. J.; Prawer, S.; Murphy, A. B. Kinetic modelling of NH_3 production in N_2–H_2 non-equilibrium atmospheric-pressure plasma catalysis. *Journal of Physics D: Applied Physics* **2017**, *50*, 154005.

(31) Fridman, A. A. *Plasma chemistry*, 1st ed.; Cambridge University Press: Cambridge, 2012.
